# A platform of serially transplantable AML PDX models covering subgroups for which no cell lines exist

**DOI:** 10.1002/hem3.70497

**Published:** 2026-10-05

**Authors:** Binje Vick, Vindi Jurinovic, Kristina Kuhbandner, Lena Lagally, Lisa Latzko, Chiara Arnreich, Gerulf Hänel, Amelie Muth, Maximilian Schönung, Arnold Kloos, Maja Rothenberg‐Thurley, Annika M. Dufour, Stephanie Schneider, Lesca M. Holdt, Liliana Mura, Fabian Klein, Annette Frank, Dhruv Mehra, Annika Fröhlich, Johannes W. Bagnoli, Maya C. André, Claudia D. Baldus, Martin Carroll, Christine Dierks, Martin Ebinger, Katharina S. Götze, Pablo Menéndez, Christian Récher, Ambrine Sahal, Jean‐Emmanuel Sarry, Christian Thiede, Talía Velasco‐Hernández, Xiaoyan Wei, Jan H. Klusmann, Wolfgang Enard, Michael von Bergwelt‐Baildon, Wolfgang Hiddemann, Klaus H. Metzeler, Philipp A. Greif, Michael Heuser, Daniel B. Lipka, Marion Subklewe, Sebastian Vosberg, Tobias Herold, Karsten Spiekermann, Irmela Jeremias

**Affiliations:** ^1^ Research Unit Apoptosis in Hematopoietic Stem Cells Helmholtz Munich, German Research Center for Environmental Health (HMGU) Munich Germany; ^2^ German Cancer Consortium (DKTK), partner site Munich, a partnership between DKFZ and University Hospital LMU Munich Munich Germany; ^3^ Institute for Medical Information Processing, Biometry, and Epidemiology (IBE) LMU Medizin, LMU Munich Munich Germany; ^4^ Department of Medicine III LMU University Hospital, LMU Medizin, LMU Munich Munich Germany; ^5^ Hopp Children's Cancer Center Heidelberg (KiTZ) Heidelberg Germany; ^6^ Manfred Fuchs Research Group Pediatric Leukemia Heidelberg University Hospital Heidelberg Germany; ^7^ Laboratory for Translational Cancer Immunology LMU Gene Center Munich Germany; ^8^ Section of Translational Cancer Epigenomics, Division of Translational Medical Oncology German Cancer Research Center (DKFZ) Heidelberg Germany; ^9^ National Center for Tumor Diseases (NCT) NCT Heidelberg, a partnership between DKFZ and Heidelberg University Hospital Heidelberg Germany; ^10^ Department of Internal Medicine IV, University Hospital Halle (Saale) Martin‐Luther‐University Halle‐Wittenberg Halle Germany; ^11^ Institute of Laboratory Medicine LMU University Hospital, LMU Medizin, LMU Munich Munich Germany; ^12^ Department of Pediatrics, Dr. von Hauner Children's Hospital LMU University Hospital, LMU Medizin, LMU Munich Munich Germany; ^13^ Anthropology & Human Genomics Faculty of Biology, LMU Munich Munich Germany; ^14^ Department of Pediatric Hematology and Oncology, University Children's Hospital Eberhard Karls University Tuebingen Germany; ^15^ Department of Internal Medicine II (Hematology/Oncology) University Hospital Schleswig‐Holstein Kiel Germany; ^16^ Division of Hematology/Oncology, Department of Medicine The Perelman School of Medicine at The University of Pennsylvania Philadelphia Pennsylvania United States; ^17^ Department of Hematology and Oncology, Freiburg University Medical Center Albert‐Ludwigs‐University of Freiburg Breisgau Germany; ^18^ German Cancer Consortium (DKTK) and German Cancer Research Center (DKFZ) Partner site Tübingen Tübingen Germany; ^19^ Department of Internal Medicine III Technical University of Munich, School of Medicine, TUM University Hospital Munich Germany; ^20^ Bavarian Cancer Research Center (BZKF) partner site Munich Munich Germany; ^21^ Josep Carreras Leukemia Research Institute Barcelona Spain; ^22^ Red Española de Terapias Avanzadas (TERAV) Instituto de Salud Carlos III Madrid Spain; ^23^ Departments of Biomedical Sciences and Medicine and Health Sciences University of Barcelona Barcelona Spain; ^24^ Centro de Investigación Biomédica en Red de Cáncer (CIBERONC) Madrid Spain; ^25^ Institució Catalana de Recerca i Estudis Avançats (ICREA) Barcelona Spain; ^26^ Pediatric Cancer Centre Barcelona‐Institut de Recerca Sant Joan de Deu (PCCB‐SJD) Barcelona Spain; ^27^ University of Toulouse Cancer Research Center of Toulouse, Inserm Toulouse France; ^28^ Equipe Labellisée LIGUE 2023 and Labex TOUCAN Toulouse France; ^29^ Université de Toulouse Centre Hospitalier Universitaire de Toulouse Toulouse France; ^30^ Department of Internal Medicine I, University Hospital Carl Gustav Carus University of Technology Dresden Germany; ^31^ Department of Pediatrics Goethe‐University Frankfurt Frankfurt Germany; ^32^ German Cancer Consortium (DKTK) DKFZ, Core Center Heidelberg Heidelberg Germany; ^33^ Present address: Department of Hematology, Oncology, and Stem‐Cell Transplantation, Munich Hospital Bogenhausen Munich Municipal Hospital Group Munich Germany; ^34^ Present address: Department for Hematology, Cell Therapy, Hemostaseology and Infectious Diseases University of Leipzig Medical Center Leipzig Germany; ^35^ Present address: Department of Hematology/Oncology and Stem Cell Transplantation, KIM IV Martin‐Luther University Halle‐Wittenberg Halle Germany; ^36^ Present address: Clinical Research Unit CATCH ALL (KFO 5010/1) funded by the Deutsche Forschungsgemeinschaft Kiel Germany; ^37^ Present address: Division of Respiratory and Critical Care Medicine University of Basel Children's Hospital Basel Switzerland; ^38^ Present address: MIVEGEC Université de Montpellier, IRD, CNRS Montpellier France; ^39^ Present address: Department of Internal Medicine III, Hematology and Oncology University Hospital Regensburg Regensburg Germany; ^40^ Present address: Comprehensive Pneumology Center Munich (CPC‐M) German Center for Lung Research (DZL) Munich Germany; ^41^ Present address: Department of Paediatric Allergology, Dr. von Hauner Children's Hospital University Hospital, LMU Munich Munich Germany

## Abstract

Acute myeloid leukemia (AML) is a highly aggressive malignancy with poor prognosis, underlining the need for suitable model systems to develop novel treatments. To complement the few existing AML cell lines and restricted primografts, we transplanted more than 100 primary AML samples into immunocompromised mice and generated 23 exceptionally robust patient‐derived xenograft (*PDX) models of AML that allow virtually unlimited serial transplantation and efficient genetic engineering. These models represent individuals of all age groups and several WHO subgroups. *PDX models substantially outperform AML cell lines in preserving leukemia biology and include AML subgroups for which no cell lines exist, such as cytogenetically normal or *IDH1/2*‐mutant AML or simultaneous *NPM1* mutation and *FLT3‐*ITD. Their resilience to freeze‐thaw cycles supports broad dissemination across research institutions through sharing via CancerModels.org. Using lentiviruses, we stably expressed luciferase for longitudinal, noninvasive, and sensitive real‐time monitoring of leukemia burden and therapeutic response *in vivo*. This approach enabled rigorously controlled preclinical studies, including phase II‐like trials, which demonstrated highly variable treatment responses between different *PDX models, mimicking the heterogeneity in patient cohorts. Long‐term treatment, including repeated cytarabine exposure over 1 year, showed a yet undescribed decrease in leukemia's proliferation rate. These insights underscore the platform's distinctive capacity to study treatment dynamics over clinically relevant timeframes. Collectively, our unique resource represents a powerful, robust, and versatile platform with strong potential to accelerate translational AML research for the ultimate benefit of patients.

## Graphical abstract

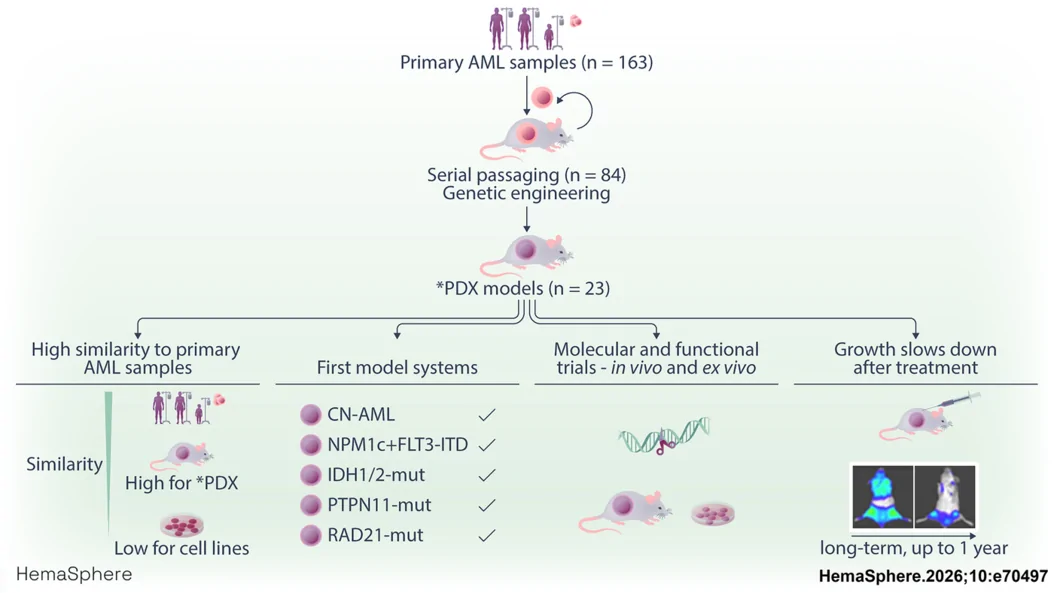

## INTRODUCTION

Acute myeloid leukemia (AML) is an aggressive and heterogenous hematologic malignancy characterized by the clonal expansion of immature myeloid cells.^1^ Initial chemotherapy can induce complete remission, but high relapse rates lead to a persistently unfavorable prognosis in AML, highlighting an urgent need for better treatment strategies.^2^ A major challenge to developing novel cancer therapies is the lack of convenient and faithful model systems. Primary patient samples are rare and often low in cell numbers, especially in children suffering from hematologic malignancies, and cells do not consistently grow *ex vivo*.^3^, ^4^ Cell lines are convenient to culture, but poorly model important features of primary tumor cells as they are heavily biased toward unstable karyotypes and certain genetic abnormalities and show altered drug response.^5^, ^6^, ^7^ While establishing a cell line from a primary tumor sample is often successful in solid tumors, this is rarely the case in liquid tumors and is often accompanied by acquired confounding features.^8^ Recent 3D organoid models of the bone marrow (BM) niche containing multiple cell types^9^ lack, for example, mechanical forces from blood flow and cannot model tumor cell migration into the BM.

Patient‐derived xenograft (PDX) models are highly attractive tools for cancer research. By transplanting primary human cancer cells into immunodeficient mice, they proliferate in an *in vivo* microenvironment. Orthotopically growing tumors, as in AML, considerably facilitate our understanding of disease pathogenesis, support reliable drug testing, and more accurately reflect the genetic and biological heterogeneity of the human disease.^10^, ^11^, ^12^, ^13^ PDXs have offered critical insights into cancer stem cell and AML biology, including mechanisms driving disease initiation, progression, and therapy resistance, (epi)genetic clonal evolution, the biology of leukemia‐initiating cells and interaction with the BM niche,^14^, ^15^, ^16^, ^17^ and many more.

Most PDX AML studies focus on primary engraftment of patient cells. A major limitation is the scarcity of primary patient material, as in certain countries only small cell numbers are collected, restricting their availability and use in repetitive assays. A decade ago, we published our first serially transplantable PDX AML models, pioneered genetic engineering of PDX AML cells, and shared the models across multiple laboratories worldwide.^18^ Building on these initial results, we have since increased the number of available models, characterized the models in depth, and were able to generate PDX models for AML subgroups for which no cell lines exist. Our cohort of serially transplantable and robust (STAR, *), genetically engineered PDX (*PDX) models of AML supports pre‐clinical studies, including phase II‐like and long‐term treatment trials, and addresses fundamental questions in leukemia pathology, ultimately facilitating the development of novel therapeutic strategies aimed at benefiting patients with the greatest unmet clinical need. They are available to the research community, and we actively encourage their broad use by sharing cells upon request. Information on the different models can be openly accessed via CancerModels.org.

## MATERIALS AND METHODS

### Mouse work

Animal trials were performed in accordance with the ethical standards of the official committee on animal experimentation and according to ARRIVE guidelines.^19^ Five‐ to 35‐week‐old male and female NOD.Cg‐Prkdc^scid^ IL2rg^tm1Wjl^/SzJ (NSG) mice (The Jackson Laboratory) were included (*n* = 2111); these were part of diverse scientific projects (see Supporting Information S1: Table S3 for a list of publications). To reduce sex bias and ensure ethical animal use, we included both female and male mice and both younger and older mice in our studies.^20^ This approach minimizes the number of animals bred without scientific purpose, in line with German regulations that prohibit the killing of animals without justified scientific need. No randomization or blinding was performed. All mice, mouse‐related interventions, and values were documented in a customized database (MyIMouse, Bioslava GbR).

### Transplantation of primary leukemia cells into NSG mice

Depending on availability of cells, 2.5E5 to 1E7 fresh or frozen/thawed primary cells were injected into the tail vein of one to eight NSG mice. In contrast to literature^16^, ^17^, ^21^, ^22^ and to avoid treatment‐induced bias, mice were not pre‐irradiated prior to cell injection. No enrichment or depletion steps were performed. Exclusion criteria were samples from EDTA or citrate‐anticoagulated material, samples with less than 25% blasts, samples with less than 2.5E5 cells, and acute promyelocytic leukemias. Information on samples is listed in Supporting Information S1: Table S1 according to PDX‐MI guidelines.^23^

### Functional scoring system of PDX models

PDX models were categorized into classes (Score 1–6) according to functional engraftment characteristics. Score 1: no primary engraftment or engraftment of B‐ or T‐cells. Score 2: primary, but no secondary engraftment. Score ≥3: serial positive engraftment; positive engraftment after freeze‐thawing cycles; engraftment primarily in BM and spleen. Score ≥4: detection of >80% human cells in BM at end‐stage leukemia; positive engraftment in at least 75% (Score 4 and 5) or at least 90% (Score 6) of transplanted mice; median time from transplantation to end‐stage leukemia below 110, 90, or 70 days (Score 4, 5, or 6, respectively). Score ≥5: successful genetic engineering. Score 6: transduction rates above 5%.

### Therapy trials

#### Repetitive treatment cycles with cytarabine (AraC) and DaunoXome (DNX)

AML‐491 *PDX cells were injected into groups of mice (5E5 luc^+^ cells/mouse). Tumor burden was regularly monitored by bioluminescence *in vivo* imaging (BLI). When BLI signals reached a total flux of around 1E9 Photons/second, mice were either treated with PBS (*n* = 7) or the first cycle of chemotherapy (C1, *n* = 15). Treated mice showed a reduction in tumor burden followed by a re‐growth of tumor cells and were again treated with a second cycle of chemotherapy (C2). When BLI signals again reached a total flux above 1E9 Photons/second, or when mice showed clinical signs of illness, the animals were sacrificed. PDX cells were re‐isolated, counted, and 5E6 cells were re‐injected into the next recipient mice. These mice received a third cycle of chemotherapy (C3, *n* = 6). Chemotherapy schedule: per cycle, three repetitions with 4 days treatment and 10 days rest; C1, Week 1 and 3: AraC 50 mg/kg d1‐4, DNX 1 mg/kg d1 + 4; C1, Week 5: AraC 100 mg/kg d1‐4; C2 and C3, Week 1, 3, and 5: AraC 100 mg/kg, d1‐4. AraC (Cell Pharma GmbH) was diluted in PBS and applied i.p., DNX (Galen Ltd.) was diluted in distilled H_2_O and applied i.v. One control mouse did not engraft reliably and was excluded from the study.

#### Long‐term treatment with azacitidine

AML‐372 (*n* = 3 per group) or AML‐491 (*n* = 4 per group) *PDX cells were injected into groups of mice (3E5 luc+ cells/mouse). Tumor burden was regularly monitored by BLI. When BLI signals reached a total flux of around 1E8 Photons/second, mice were treated i.p. either with PBS or azacitidine (2.5 mg/kg or 5 mg/kg, diluted in PBS) three times a week for 11 weeks. Monitoring of tumor burden was continued after the end of therapy.

### Enrichment of AML PDX cells for sequencing

AML PDX cells were enriched from murine BM at end‐stage leukemia via negative selection by magnetic cell sorting using a mouse cell depletion kit (Miltenyi Biotech). 100 μL MicroBeads were used per 1E7 BM cells. Enriched PDX cells were lysed in RLT‐Buffer (Qiagen) with 1% beta‐mercaptoethanol.

### Prediction models

To develop a prediction model for positive engraftment, a backward selection based on the Akaike information criterion was performed. Variables were included that had less than 10% of missing values and a univariable P ≤ 0.1.

### Statistics

Statistical analyses were performed using GraphPad Prism 10 software (Graphpad Prism) and R.^24^ Analyses used are indicated in the figure legends. n.s.: not significant; *p < 0.05, **p < 0.01, ***p < 0.001, ****p < 0.0001.

## RESULTS

### Primary AML engraftment in NSG mice is favored by aggressive disease and female mice

Primary samples of 102 adult AML patients were transplanted into mice over a period of 12 years (Supporting Information S1: Table S1); of these, 75 samples were collected at the University Hospital at LMU Munich (cohort 1, Supporting Information S1: Table S2), and 27 samples were collected either at hospitals in Germany in the framework of the German Cancer Consortium (*n* = 22) or at the hospital of the University of Pennsylvania (*n* = 5) (together cohort 2). Furthermore, PDX models were included which have been established by collaboration partners (*n* = 22), among them five from pediatric patients (cohort 3). In an independent approach, PDX models of seven AML samples from children, mainly with *KMT2A*‐rearranged AML, and of 32 AML samples from adults were established at Hannover Medical School (cohorts 4 and 5, respectively). In total, cells from 163 patients were included in the study (Figure 1A and Supporting Information S1: Table S1). Using nonirradiated NSG mice and intravenous injection, 43% and 56% of primary samples engrafted in cohorts 1 and 2, respectively, in line with published data (Figure 1B).^25^, ^26^

**Figure 1:**
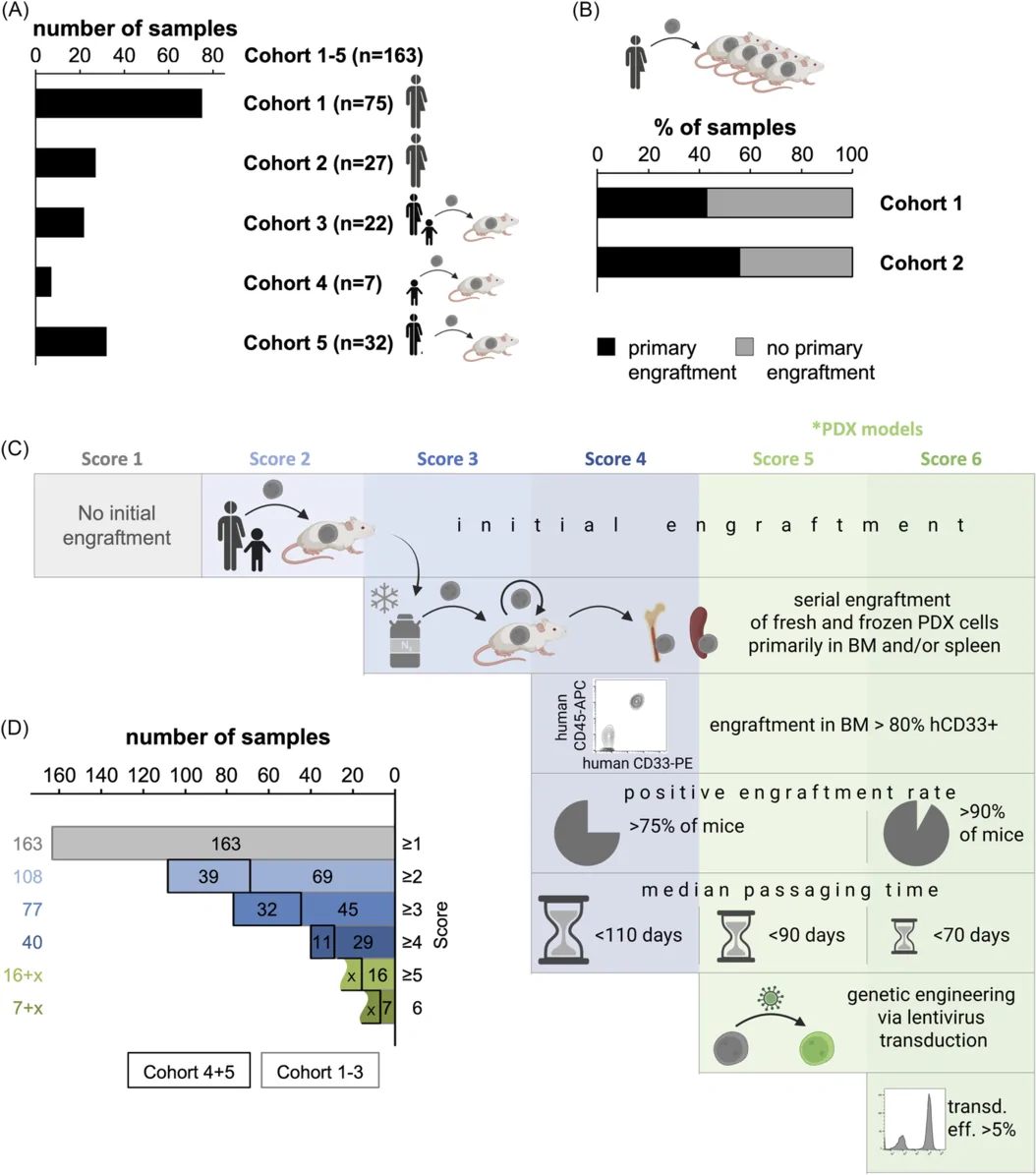
**Robust serial engraftment is confined to a minority of PDX models**. **(A)** Overview of included cohorts: cohort 1: primary samples of adult patients (*n* = 75, 34% R/R); cohort 2: primary samples of adult patients (*n* = 27, 57% R/R); cohort 3: xenografted models of adult or pediatric patients (*n* = 22, 32% R/R); cohort 4: xenografted models of pediatric patients (*n* = 7, 0% R/R); cohort 5: xenografted models of adult patients (*n* = 32, 0% R/R). R/R: relapsed/primary refractory. **(B)** Primary patient cells of cohorts 1 and 2 were transplanted into one to eight NSG mice via tail vein injection (2.5E5–2E7 cells per mouse), and positive engraftment was analyzed. If at least one mouse showed positive engraftment, the sample was classified as engrafted. **(C**, **D)** Primary AML samples (cohorts 1 and 2) and AML PDX models (cohorts 3–5) were serially transplanted into NSG mice, and positive engraftment was analyzed. Scheme illustrating the scoring system based on specific features **(C)**. Number of models within Score 2–6 **(D)**. As models of cohorts 4 and 5 have not been genetically engineered, no discrimination between scores 4, 5, or 6 is feasible.

To assess predictive parameters for primary engraftment, we analyzed concomitant clinical data available for cohort 1 and reproduced known prediction factors such as relapse/primary refractory (R/R) disease and presence of *FLT3*‐ITD mutations (Supporting Information S1: Table S2 and Supporting Information S1: Figure S1A).^22^, ^25^, ^27^, ^28^, ^29^, ^30^ Additionally, *BCOR*‐mutated samples, although low in numbers, showed a trend toward higher engraftment capacity (Supporting Information S1: Figure S1A). It has been previously shown that xenograft potential is negatively associated with overall survival (OS) and event‐free survival probability.^27^, ^28^ A similar trend was observed in cohort 1; however, the differences did not reach statistical significance, presumably due to the small sample size (Supporting Information S1: Figure S1B). This is consistent with the extremely poor prognosis for patients with relapsed disease, for example, after allogeneic BM transplant, when treatment may focus on supportive care.

Conflicting results have been published concerning engraftment capacity in male versus female NSG mice.^31^, ^32^ In our study, primary cells from male patients transplanted into male mice showed the lowest engraftment rates, while primary cells from female patients transplanted into female mice showed the highest engraftment rates; age of recipient mice, however, did not affect engraftment efficiency (Supporting Information S1: Figure S1C,D).

### A rare subfraction of primografts allows generation of PDX models with robust serial engraftment and genetic engineering

Serial re‐transplantation of PDX AML cells beyond two passages has been scarcely studied, except for our own previous data.^16^, ^18^, ^30^, ^33^, ^34^ Here, we aimed to establish robust, serially transplantable, and rapidly engrafting PDX AML models which allow freeze/thaw cycles and genetic engineering as new tools for complex downstream applications *ex vivo* and *in vivo*. To identify appropriate samples, we defined subjective characteristics based on prior experience to categorize PDX AML models into six groups, considering the following features: (i) positive primary engraftment, (ii) serial engraftment capacity of frozen/thawed cells, (iii) high engraftment rate in the BM and in a majority of mice, (iv) passaging time (period from transplantation to end‐stage leukemia) below 16 weeks, and (v) ability for lentiviral transduction (Figure 1C). Of the 108 AML samples with primary engraftment (score ≥2), only 77 (71%) showed positive serial engraftment (score ≥3). This contrasts with the closely related disease acute lymphoblastic leukemia (ALL), where both primary and serial engraftment are feasible for virtually all samples.^35^ Robust and fast serial engraftment was achieved in 40/77 samples (52%, score ≥4). While genetic manipulation was not conducted in samples of cohorts 4 and 5, 23/29 (79%) Score 4 samples of cohorts 1‐3 allowed genetic engineering (score ≥5, Figure 1C,D and Supporting Information S1: Table S1E). We called those score 5/6 samples *PDX models of AML to indicate their ability for serial transplantation and robust engraftment (STAR) and genetic engineering. In summary, *PDX models were generated from only around one‐fourth of primografts.

### *In vivo* characteristics of *PDX models

Individual *PDX models showed re‐engraftment in up to 200 mice, up to 18 consecutive passages, and thereby *in vivo* growth for up to 800 consecutive days until today, indicating the presence of nonexhausting leukemia‐initiating cells (Supporting Information S1: Table S1 and Supporting Information S1: Figure S1F,G). The passaging time was highly heterogeneous between and within models, with median passaging times ranging from 42 to 105 days, dependent on injected cell numbers and mouse sex, and other factors in individual models (Supporting Information S1: Figure S1H,I). The positive engraftment rate (PER) remained constant over sequential passaging and was independent of the number of injected cells, use of freshly isolated versus thawed cells, or recipient mouse age for most models (Supporting Information S1: Figure S1J). On purpose, we included male and female mice in our studies to prevent sex bias and ensure ethical animal use. While genetic engineering mildly affected PER, mouse sex had a more pronounced effect, with higher PER in female than in male mice in some models, in line with published data (Supporting Information S1: Figure S1J,K).^32^ PER was slightly reduced in old mice (≥20 weeks) compared to young mice (≤10 weeks) (Supporting Information S1: Figure S1L).

The amount of PDX cells that could be re‐isolated from BM or spleen at end‐stage leukemia was highly heterogenous within and across *PDX models; splenic engraftment associated with splenomegaly was observed in a subfraction and at late disease stages (Supporting Information S1: Figure S1M), while we could not define any factors predicting splenic involvement (data not shown). Percentage of PDX cells in PB at end‐stage leukemia differed between the models (Supporting Information S1: Figure S1N). A particular advantage of the model is that, upon careful observation, most experiments can be terminated with mice showing no or only mild symptoms (Supporting Information S1: Figure S1O).

### *PDX models represent aggressive diseases of diverse subgroups

*PDX models might represent a biased subfraction of AML in patients; thus, we next examined variables associated with these models. As for primary engraftment (Supporting Information S1: Figure S1A), disease status emerged as the most robust predictor for the ability to generate *PDX models (Figure 2A and Supporting Information S1: Figure S2A); accordingly, patients whose cells gave rise to a *PDX model showed dismal OS (Figure 2B and Supporting Information S1: Figure S2B) and thus represent a population of high need for better treatment. Extramedullary disease (EMD) is another feature of aggressive leukemia and is present in about 2.5%–9% of AML patients at initial diagnosis or 10% after allogeneic stem cell transplantation.^37^, ^38^ We found that 4/23 (17%) *PDX models and 7/29 (25%) with scores 3 to 6 were generated from patients with EMD. Of note, the 23 *PDX models were retrieved from patients of all age groups and from both sexes and originated from five distinct AML World Health Organization (WHO) subgroups at representative proportions (Figure 2C).^39^, ^40^, ^41^, ^42^, ^43^

**Figure 2:**
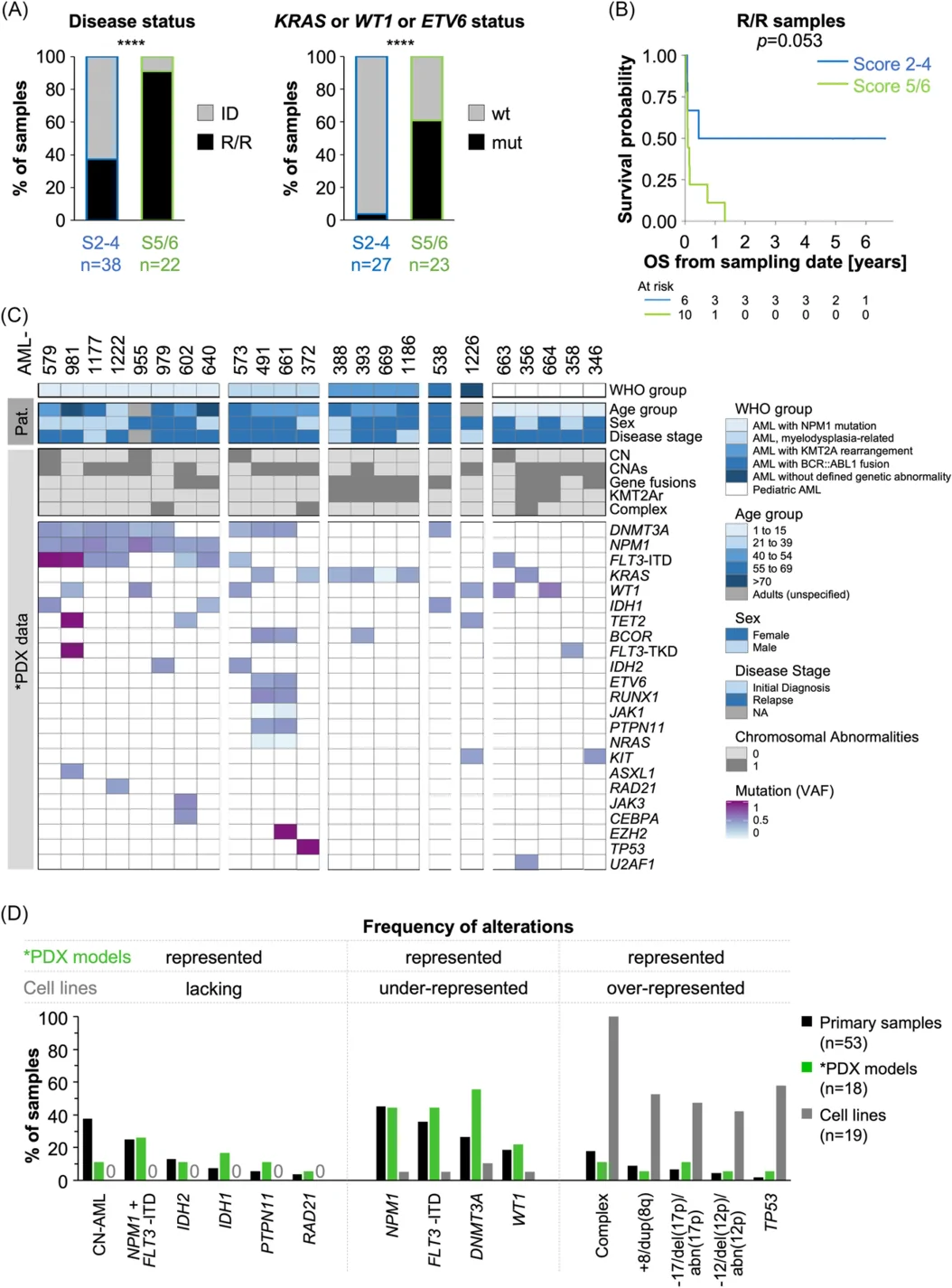
*PDX models represent aggressive diseases of diverse AML subgroups and resemble primary AML cells better than cell lines. **(A)** Prediction model for Score 5/6 (*n* = 23) versus Score 2–4 (*n* = 46) was calculated by backward selection based on the Akaike information criterion, including samples of cohort 1–3. Left: percentage of models originating from initial diagnosis (ID, gray) or primary refractory/relapse (R/R, black) patient samples are depicted (P = 8.7E−4). Right: percentage of models with no (gray) or detected mutation in *KRAS* or *WT1* or *ETV6* (black) are depicted (P = 1.8E−05). The P‐values were calculated with Fisher's exact test. **(B)** Kaplan–Meier graph showing the survival probability of patients with R/R disease in cohort 1 whose cells produced Score 5/6 PDX models (green) or did not meet the criteria for high‐score models (Score 2–4, blue). Log‐rank test, P = 0.053. (**C)** Clinical features and genomic landscape of *PDX models. *PDX models are presented and categorized according to their WHO group. Patient (Pat.) data indicate age group, sex, and disease status at the time of sample collection. *PDX models were characterized by cytogenetics, low‐coverage whole‐genome sequencing, RNA sequencing, methylation profiling, and DNA panel sequencing. PDX data depict cytogenetically normal (CN) models, detection of copy number alterations (CNAs), gene fusions, *KMT2A* rearrangements, and complex karyotype, as identified with at least two independent methods. Recurrent mutations are presented by variant allele frequency (VAF). **(D)** Relative frequencies of samples/models with specific mutational or chromosomal alterations are depicted for primary adult patient samples at relapsed disease of the BEAT cohort (*n* = 53, black), *PDX models generated from adult patients (*n* = 18, green), and AML cell lines^36^ generated from adult patients and having more than 500 hits in Google Scholar (*n* = 19, gray). Events are included if they are present in a minimum of 5% in one of the three cohorts and are found in primary patients. Alterations are categorized according to prevalence in cell line models and sorted according to relative frequencies in patients. Refer to Supporting Information S1: Figure S2J for further categories.

### *PDX models resemble primary AML better than cell lines

Molecular alterations within serially passaged *PDX cells were studied using whole‐genome sequencing, panel sequencing, full‐length RNA sequencing, cytogenetics, methylation profiling, and fluorescence in situ hybridization (Supporting Information S1: Table S1). At the genomic level and in contrast to primary engraftment, mutations in *WT1*, *KRAS,* or *ETV6*, but not in *FLT3‐*ITD or *BCOR*, were associated with an increased likelihood of generating *PDX models (Figure 2A and Supporting Information S1: Figure S2C), although the presence of *WT1*, *KRAS,* or *ETV6* mutations does not correlate with patient OS probability (Supporting Information S1: Figure S2D).

Our *PDX models exhibited a wide range of chromosomal alterations and/or mutations known to be associated with AML (Figure 2C and Supporting Information S1: Table S1). Longitudinal analysis of variant allele frequencies (VAF) across serial passages revealed that clones defined by AML‐specific mutations remain largely stable over time, consistent with our own previous data.^18^ The most pronounced changes in clonal composition occurred during primary engraftment, in line with published data^17^, ^44^, ^45^ (Supporting Information S1: Figure S2E,F,G). Furthermore, transcriptome analysis revealed the presence of both known fusions and undescribed fusion genes (Figure 2C and Supporting Information S1: Figure S2H,I; Supporting Information S1: Table S1).

We compared the frequency of alterations between primary AML cells, *PDX models, and cell lines. In the initial analysis, we included primary AML samples from adult patients at relapse from the BEAT cohort (*n* = 53),^46^ *PDX models from adults (*n* = 18), and AML cell lines from adults which were most frequently used in research (*n* = 19).^36^

Importantly, cytogenetically normal samples, samples with both *NPM1* mutation and *FLT3*‐ITD, and mutations in *IDH1*, *IDH2*, *PTPN11,* and *RAD21* can be modeled in *PDX models but are absent in AML cell lines or in most frequently applied cell lines, respectively. Additionally, mutations most prevalent in primary patient cells—*NPM1*, *FLT3‐*ITD, *DNMT3A,* and *WT1*—are recapitulated in *PDX models at similar frequencies but are severely underrepresented in cell lines. Conversely, cell lines have a strong bias toward *TP53* mutations and complex karyotypes, while primary samples and *PDX models display these alterations at low frequencies (Figure 2D and Supporting Information S1: Figure S2K,L). Several alterations were represented by both *PDX models and cell lines, with *PDX models overrepresenting *KRAS* mutations and *KMT2A* rearrangements. Some rare AML mutations were not found in *PDX models, explainable by the limited number of primary samples studied. Unfortunately, some mutations are still not represented, neither in *PDX models nor in cell lines, for example, biallelic *CEBPA, SF3B1,* or *SRSF2*, although for the latter we were able to establish serially transplantable, although less robust PDX models of score 3 (Supporting Information S1: Figure S2J–L and Supporting Information S1: Table S1).

When we further broadened the analyses by including additional primary patient data sets,^47^, ^48^ scores 3–6 AML PDX models derived from adult patients (*n* = 45), and all adult AML cell lines (*n* = 54),^36^ we could reproduce the results described above and additionally found that the number of AML‐specific mutations was similar in PDX models and primary samples (Supporting Information S1: Figure S2L,M). In summary, PDX models were clearly superior to cell lines in mimicking the genetic and chromosomal alterations found in primary AML cells, including alterations for which no cell lines exist.

Next, we analyzed DNA methylation by bisulfite sequencing and gene expression by 3′‐tag RNA‐sequencing^49^ from PDX models (Supporting Information S1: Table S1) and compared them to available own data from AML patient samples at initial diagnosis (AML‐CG Registry, *n* = 224).^50^, ^51^ Mutation prediction models trained on transcriptome and methylome data of primary patient samples showed comparable performance in patient and PDX cohorts (Figure 3A and Supporting Information S1: Figure S3A). This suggests that the transcriptional programs and methylation patterns associated with recurrent AML mutations are largely preserved in PDX samples. To study epigenetic consistency of *PDX models compared to primary AML cells, a recently established DNA methylation‐based hierarchical classification framework was used.^52^ This framework predicts disease entities and epigenetic disease subgroups (epitypes) from DNA methylation data. All samples were correctly classified as AML, while AML epitypes were successfully determined for 17/25 (68%) models.^53^ Of these, genetic mutations that are characteristic of the respective epitype were detected in genomic analysis of 15 samples (88%), indicating a high concordance of both readout layers. The remaining eight models did not pass the epitype prediction threshold, whereby still in 7/8 models, measured genotypes matched low‐confidence epitype predictions (Figure 3B).

**Figure 3:**
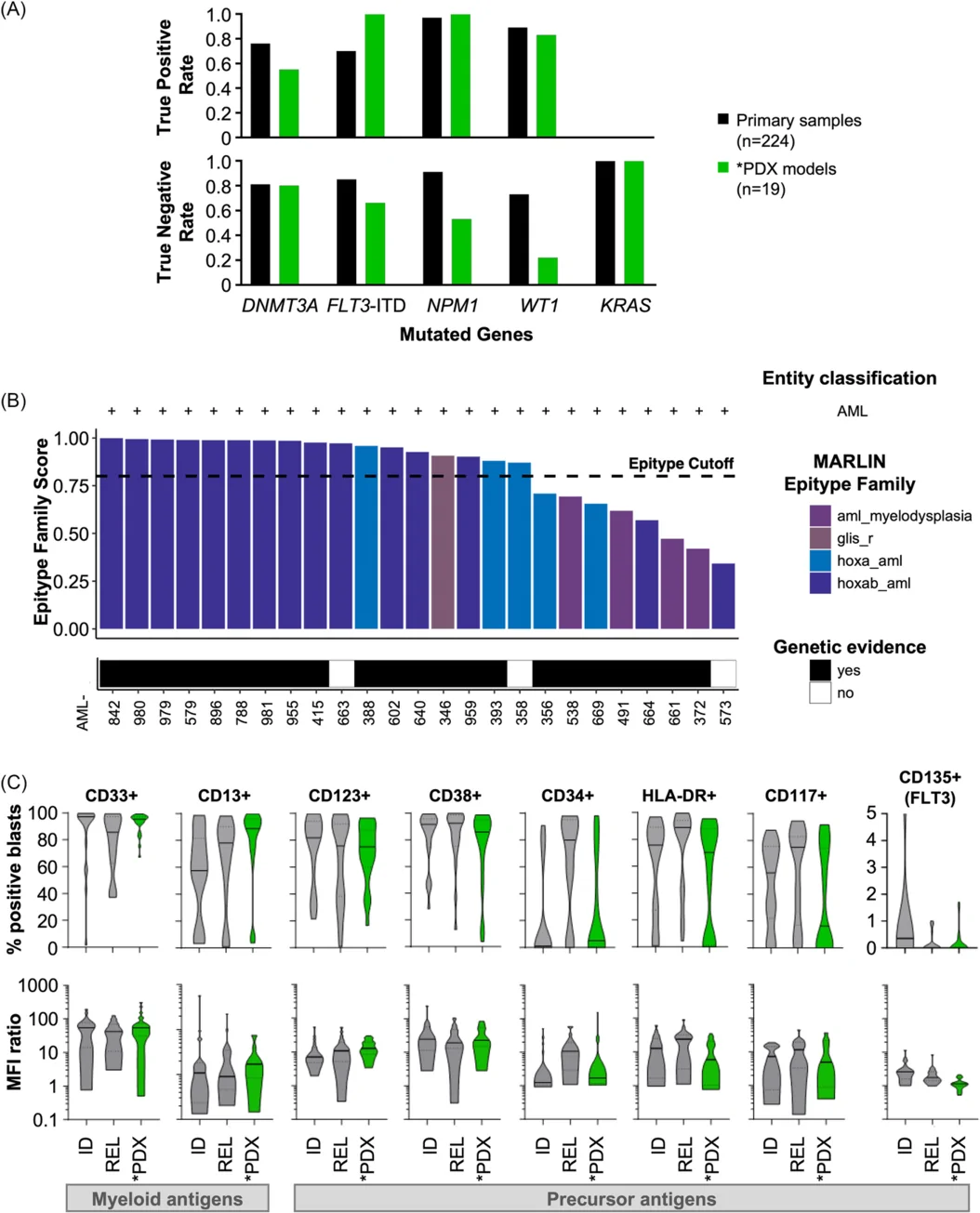
*PDX models resemble primary AML cells. **(A)** Prediction analysis of microarrays (PAM) classifiers based on DNA methylation profiling evaluated on AML‐CG Registry patient samples (*n* = 224) and *PDX models (*n* = 19). Displayed are the true positive rate and true negative rate for genes mutated in at least 20% of PDX samples. **(B)** DNA methylation‐based disease classification. Hierarchical classification was applied to PDX AML models (*n* = 25). Data show successful classification according to disease entity, MARLIN epitype family classification, and genetic evidence. **(C)** Immunophenotype of *PDX models resembles primary patient samples. Primary patient samples at initial diagnosis (ID, *n* = 20), primary patient samples at relapse (REL, *n* = 20), and *PDX models (*n* = 23, green) were analyzed for surface molecule expression via flow cytometry. Primary patient samples originated from an independent cohort. Violin plots depicting the percentage of antigen‐positive cells within the blast gate (upper) or mean fluorescence intensity (MFI, lower); median indicated by solid line and 25th and 75th percentile by dotted lines.

Finally, *PDX models maintained key immunophenotypic characteristics of primary AML cells, expressing myeloid and precursor antigens at heterogeneous levels and in similar percentages across all cohorts (CD33, CD123, and HLA‐DR) or more biased toward samples from initial diagnosis (CD34 and CD33/CD7) or relapse (CD135 (FLT3) and CD33/CD64) (Figure 3C and Supporting Information S1: Figure S3B,C,D). In summary, *PDX models largely mimic primary patient AML cells at the level of genome, transcriptome, methylome, and immunophenotype.

### *PDX models allow a wide range of *ex vivo* and *in vivo* experiments

Until today, *PDX models have been successfully used for a variety of applications, including functional and molecular assays and treatment trials, and were included in 48 scientific publications from collaboration partners across the globe and us (Figure 4A and Supporting Information S1: Figure S4A–C; see Supporting Information S1: Table S3 for a list of publications). To actively encourage their broader scientific use, these models were included in the openly accessible repository CancerModels.org, and cells will be shared upon request. For cutting‐edge *in vivo* experiments, *PDX models were genetically engineered to stably express enhanced firefly luciferase for sensitive and repetitive monitoring of tumor burden using BLI.^18^, ^54^ For molecular studies, derivative *PDX models were generated which express Cre‐ER^T2^ or Cas9, enabling *in vivo* inducible shRNA knockdown or CRISPR‐mediated gene knockout, respectively^55^, ^56^, ^57^ (Supporting Information S1: Figure S4D and Supporting Information S1: Table S3). *PDX cells freshly isolated from donor mice display higher viability than frozen/thawed cells, allowing complex *ex vivo* studies with high cell numbers and over several weeks (Supporting Information S1: Figure S4E). As prolonged *ex vivo* culture induced reversible changes in the immunophenotype of *PDX cells, most prominently in AML‐388 (Supporting Information S1: Figure S4F), studies were preferably performed using *PDX cells freshly isolated from donor mice.

**Figure 4:**
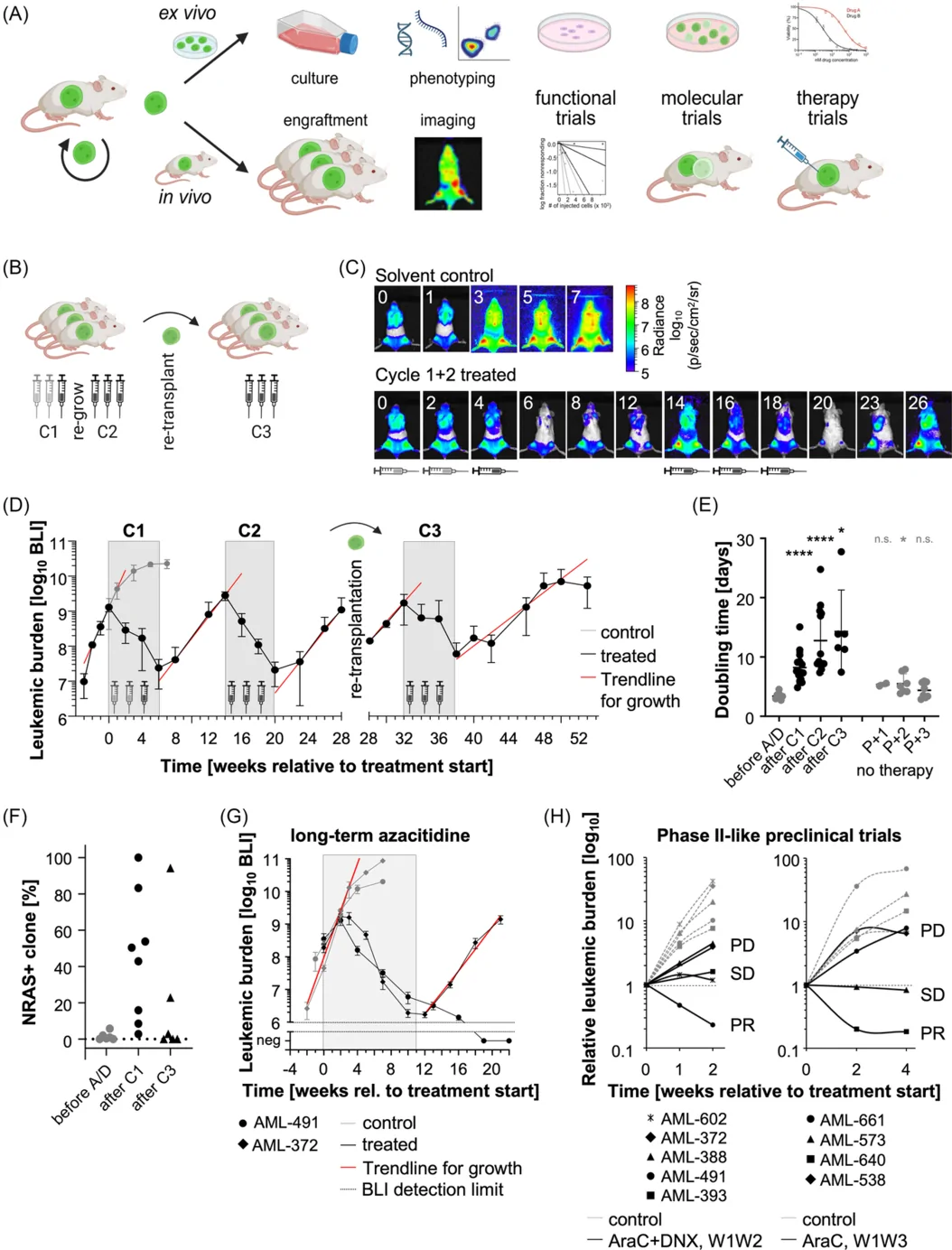
*PDX models allow a wide range of ***ex vivo*** **and** ***in vivo*** **experiments, including long‐term** ***in vivo*** **treatment trials**. **(A)** Scheme illustrating published *in vivo* and *ex vivo* trials conducted with *PDX models. For a list of respective publications, see Supporting Information S1: Table S3. **(B**–**F)** Repetitive treatment to model induction plus consolidation chemotherapy. *PDX AML‐491 cells were injected into mice and tumor burden was monitored by BLI. At a total flux of around 1E9 P/s, mice were either treated with PBS (*n* = 7, gray) or two cycles of chemotherapy (C1 and C2, *n* = 15, black). After re‐growth, animals were sacrificed, and PDX cells were re‐injected into the next recipient mice receiving a third cycle of chemotherapy (C3, *n* = 6). **(B)** Scheme. Gray syringe: AraC 50 mg/kg, d1 − 4 + DNX 1 mg/kg, d1 + 4, 10 days rest; black syringe: AraC 100 mg/kg, d1 − 4, 10 days rest. **(C)** BLI pictures of two exemplary mice are shown; numbers indicate weeks after treatment start. **(D)** Quantification of BLI. Gray box indicates the treatment period, and red lines show the exponential trend line during growth. **(E)** Doubling time, measured by increase in BLI signal, is depicted for untreated mice (before A/D, *n* = 7), after stop of 1st (C1, *n* = 15), 2nd (C2, *n* = 13), or 3rd (C3, *n* = 6) cycle of chemotherapy, or of untreated cells after 1, 2, or 3 re‐transplantation steps (P + 1 *n* = 2, P + 2 *n* = 7, P + 3 *n* = 8). Dots indicate values of individual mice; the line indicates the median. n.s. not significant, *P < 0.05, ****P < 0.0001 as tested by two‐sided unpaired *t*‐test with Welch's correction. **(F)** Panel sequencing was performed with re‐isolated PDX cells. Percentage of NRAS‐mutant cells is depicted. **(G)** Long‐term therapy with azacitidine. AML‐372 (*n* = 4–5 per group, diamonds) or AML‐491 (*n* = 4 per group, circles) *PDX cells were injected into mice and tumor burden was monitored by BLI. Mice were either treated with PBS (gray) or azacitidine (black) for 11 weeks. Quantification of BLI. Gray box indicates the treatment period, and the red lines show the exponential trend line during growth. **(H)** Phase II‐like preclinical trials. Diverse *PDX models were injected into mice and tumor burden was monitored by BLI. Mice were treated with PBS (gray) or AraC+DNX for two consecutive weeks (left) or with AraC in Week 1 and Week 3 (right, black); AraC 100 mg/kg, d1 − 4, DNX 1 mg/kg, d1 + 4. Relative tumor burden compared to the start of therapy was calculated; the mean is indicated for each model. Left: AML‐602 (stars, *n* = 5), AML‐372 (diamonds, *n* = 4), AML‐388 (triangles, *n* = 4), AML‐491 (circles, *n* = 7), and AML‐393 (squares, *n* = 6); right: AML‐661 (circles, *n* = 6), AML‐573 (triangles, *n* = 3), AML‐640 (squares, *n* = 3), and AML‐538 (diamonds, *n* = 4). PD, progressive disease; PR, partial response; SD, stable disease.

### Long‐term *in vivo* experiments show reduced growth rates after treatment

As an example of their application, we show here that *PDX models and repetitive *in vivo* imaging enabled long‐term *in vivo* treatment trials and thus provide new insights into disease biology. Spontaneous tumor (re‐) growth cannot be directly studied in leukemia patients due to the necessity of immediate and ongoing treatment following diagnosis. This obscures our understanding of how treatment influences natural tumor progression. While accelerated growth following chemotherapy has been reported for certain solid tumors, leukemia growth dynamics remain largely unexplored.^58^, ^59^

Using an adapted form of a mouse equivalent to the “7+3” induction therapy in patients,^60^, ^61^ we mimicked induction‐ and consolidation‐therapy and treated mice carrying luciferase‐transgenic *PDX AML‐491 cells with repetitive cycles of chemotherapy (Figure 4B–F); despite expected reduction in blood cell counts,^62^ the treatment was well tolerated over prolonged periods of time (Supporting Information S1: Figure S4G,H). After 1 year of treatment with nine blocks of chemotherapy in three cycles, and a re‐transplantation to respect mouse wellbeing, we were surprised to find that response to treatment remained unchanged, with no signs of acquired resistance. However, tumor growth rate decreased continuously with each treatment block, but not in untreated controls (Figure 4D,E and Supporting Information S1: Figure S4I). Therapy selected for a minor NRAS‐mutated clone in some mice, which did not show any differences in response or growth rate compared to the NRAS^wt^ clone (Figure 4F and Supporting Information S1: Figure S4J).^63^

To validate this finding in a different setting, we studied hypomethylating agents that are routinely used to treat less fit AML patients.^64^ Mice carrying *TP53*‐mutant *PDX AML‐372 showed tumor re‐growth after the end of azacitidine treatment with an increase in doubling time, indicating that a reduced growth rate after treatment represents a shared feature across different treatment modalities and across different *PDX models (Figure 4G and Supporting Information S1: Figure S5K‐M).

Thus, our *PDX models allowed insights into cancer biology that are inaccessible in patients. In contrast to data published for solid tumors,^58^, ^59^ we found a reduced *in vivo* growth rate after treatment, which is consistent with the known higher efficacy of chemotherapy against dividing tumor cells compared to non‐dividing ones.

### *PDX models allow phase II‐like preclinical trials

Preclinical trials in mouse models are an essential step in the drug development pipeline prior to testing drugs in humans. However, the majority of clinically tested drugs fail to reach approval and routine clinical application due to limited efficacy or toxicity.^65^ One promising strategy to overcome the limitations of conventional preclinical studies is conducting phase II‐like preclinical trials which enable adequately powered studies to assess drug efficacy across different tumor subgroups.^13^, ^33^ Mimicking challenges in the clinics, such preclinical trials allow studying the heterogeneity of response of different patient tumors to the same treatment as well as of a single patient's tumor to different treatments. Of note, while phase II‐like trials are more frequently implemented for disease entities such as ALL,^33^, ^66^ these studies remain very limited in AML due to the high cell numbers required for primograft experiments.

When mice transplanted with *PDX models were treated with two different regimens of conventional chemotherapy, we observed varying treatment responses for individual *PDX models, including complete response, stable disease, and progressive disease (Figure 4H and Supporting Information S1: Figure S4N). Notably, treatment responses observed in our *PDX models *in vivo* closely mirrored the clinical outcomes of the corresponding patients, underscoring the translational relevance of our platform. Patient AML‐491 received cytarabine‐based therapy (including cytarabinosid s.c., cytarabin, hydroxycarbamid, and clofarabin) prior to allogeneic stem cell transplantation and survived 484 days after sampling. Consistent with this favorable clinical response, AML‐491 *PDX cells demonstrated sensitivity to cytarabine and DaunoXome *in vivo*.^63^, ^67^ In contrast, patients AML‐372, AML‐388, and AML‐393 were refractory to intensive chemotherapy containing cytarabine and exhibited poor clinical outcomes, surviving only 57, 28, or 25 days after sampling, respectively. *PDX cells of AML‐372, AML‐388, and AML‐393 faithfully recapitulated this resistant phenotype, displaying progressive disease in treated mice (Figure 4H and Supporting Information S1: Figure S4N). Of note, serial passaging and genetic engineering did not affect therapy response *ex vivo* (Figure S4O–R).

Our established cohort of *PDX models thus presents an ideal tool to test novel therapies on cohorts of AML patient tumors in reliable, sensitive, and rigorously controlled preclinical treatment trials (see Supporting Information S1: Table S3 for a list of publications). As models are shared via CancerModels.org, they can be used in laboratories worldwide.

In summary, we established *PDX models—serially transplantable and robust genetically engineered PDX models of AML—for advanced translational and preclinical trials, harboring the potential to foster future AML research for the ultimate benefit of patients suffering from AML.

## DISCUSSION

To advance cancer research, we established and characterized *PDX models as a broadly accessible and shareable tool according to PDX‐MI guidelines.^23^ They fulfill clearly defined criteria regarding the ability of serial transplantation, robust engraftment, and genetic engineering. *PDX models represent alterations found in primary AML patient cells, both at initial diagnosis and relapse, and include subgroups that cannot be modeled with cell lines, such as CN AML, *IDH1/2* mutations, or co‐occurring *NPM1* and *FLT3‐*ITD mutations. They are used for complex molecular and functional studies, both *ex vivo* and *in vivo*, and are especially suitable for long‐term preclinical treatment trials.

*PDX models both complement and, in several key aspects, outperform existing model systems such as primary tumor cells, primografts, and cell lines. Primary patient cells and primografts are typically available mostly in limited numbers, especially from pediatric patients and in certain countries, and are rarely shared, thus complicating data reproducibility and standardization of experiments. However, it has already been extensively described that primografts undergo clonal selection favoring “relapse‐committed” clones,^17^, ^44^, ^45^, ^68^ thus complicating co‐clinical trials for individual patients. Additionally, clones might be further enriched during serial passaging.^28^ Nevertheless, our *PDX models, even from advanced passages and after genetic engineering, showed high overall concordance with primary patient samples regarding mutational landscape, chromosomal alterations, epitypes, and immunophenotype. As shown before, neither serial passaging nor genetic engineering had a major impact on characteristics of *PDX models, including mutations, transcriptome, or therapy response.^18^, ^49^, ^69^ As an additional advantage, virtually endless serial transplantation and amplification facilitate the application of *PDX AML models similar to AML cell lines as highly valuable, shareable tools for research, for example, in subgroups for which no cell lines exist.

*PDX models are biased toward high‐risk subgroups. Primary leukemia cells from AML subtypes with less dismal prognosis showed positive primary engraftment in immunocompromised mice only after long latency and at low percentage,^22^ making it impossible to develop *PDX models as defined here. The advantage of *PDX models lies in their ability to provide highly patient‐related cells repetitively and reproducibly for a wide variety of different AML subtypes and clones across the broad heterogeneity of AML. This also warrants their use in larger, phase II‐like preclinical trials, mimicking human randomized clinical trials. Such an approach offers a promising strategy to enhance the rate of successful translation into clinical practice by enabling adequately powered studies to assess drug efficacy across different tumor subgroups.^13^, ^33^ Our findings indicate that our models could help to identify patient subsets most likely to benefit from specific therapies, enabling targeted pre‐selection for inclusion in subsequent clinical trials.

Compared to cell lines, PDX models are time‐ and resource‐intensive and logistically complex.^70^, ^71^ Nevertheless and importantly, PDX models of AML harbor major advantages over cell lines which include (i) orthotopic engraftment due to a persistent dependence on the physiologic tumor microenvironment^67^, ^72^; (ii) maintained stem cell hierarchy^18^, ^73^, ^74^; (iii) higher similarity to primary AML cells; (iv) availability of AML subtypes for which no cell lines exist; and (v) improved predictive value for drug responses.^62^, ^75^

*PDX models allow performing prolonged *in vivo* treatment trials. Repetitive *in vivo* imaging facilitates close monitoring of tumor burden under treatment and thereby enabled, to our knowledge, the first preclinical *in vivo* trial in acute leukemia which continued for 12 consecutive months. The technical advance allowed studying the kinetics of tumor repopulation after treatment, a topic that was often neglected so far, but is relevant to understanding real‐world treatment outcomes.^59^ In contrast to findings in solid tumors, where tumor growth was accelerated after chemotherapy,^58^, ^59^ we observed slower growth after treatment. Our results are consistent with the well‐known fact that chemotherapy is more effective in proliferating cells than in resting cells.^67^, ^76^ Of relevance for clinical decision‐making, our data show that AML growth dynamics might substantially differ directly after treatment compared to disease relapse. Of note, we have previously observed a significant increase in doubling time of a long‐term chemotherapy‐treated ALL PDX model compared to treatment‐naïve cells upon re‐transplantation.^77^ Together, these findings suggest that slowed tumor growth after chemotherapy might be a special feature of acute leukemia and highlight the platform's suitability for modeling treatment dynamics and tumor cell adaptation.

In summary, *PDX models mimic the human disease more closely than cell lines and provide a reliable preclinical platform for testing therapies, including novel treatment strategies, across multiple genetically distinct AML subtypes, generating data that can inform subsequent clinical trials. We envision *PDX models being globally shared, analogous to cell lines, driving scientific progress and ultimately contributing to improved prognosis for patients suffering from AML.

## AUTHOR CONTRIBUTIONS

**Binje Vick**: Conceptualization; Investigation; Writing—original draft; Writing—review and editing; Visualization; Validation; Methodology; Formal analysis; Project administration; Data curation; Supervision. **Vindi Jurinovic**: Formal analysis; Data curation; Supervision; Visualization; Writing—review and editing; Methodology; Investigation; Validation. **Kristina Kuhbandner**: Writing—original draft; Writing—review and editing; Visualization; Conceptualization. **Lena Lagally**: Formal analysis; Visualization; Methodology. **Lisa Latzko**: Formal analysis; Methodology; Visualization. **Chiara Arnreich**: Data curation; Formal analysis. **Gerulf Hänel**: Formal analysis; Data curation. **Amelie Muth**: Formal analysis; Data curation. **Maximilian Schönung**: Methodology; Formal analysis. **Arnold Kloos**: Resources; Investigation. **Maja Rothenberg‐Thurley**: Data curation; Formal analysis; Methodology. **Annika M. Dufour**: Data curation; Formal analysis. **Stephanie Schneider**: Data curation; Supervision. **Lesca M. Holdt**: Data curation; Formal analysis; Methodology. **Liliana Mura**: Investigation; Project administration. **Fabian Klein**: Investigation; Software; Data curation; Formal analysis. **Annette Frank**: Investigation; Formal analysis. **Dhruv Mehra**: Investigation. **Annika Fröhlich**: Investigation. **Johannes W. Bagnoli**: Investigation; Data curation; Formal analysis; Methodology. **Maya C. André**: Resources. **Claudia D. Baldus**: Resources. **Martin Carroll**: Resources. **Christine Dierks**: Resources. **Martin Ebinger**: Resources. **Katharina S. Götze**: Resources. **Pablo Menéndez**: Resources. **Christian Récher**: Resources. **Ambrine Sahal**: Resources. **Jean‐Emmanuel Sarry**: Resources. **Christian Thiede**: Resources. **Talía Velasco‐Hernández**: Resources. **Xiaoyan Wei**: Resources. **Jan H. Klusmann**: Resources. **Wolfgang Enard**: Methodology; Supervision. **Michael von Bergwelt‐Baildon**: Supervision; Resources. **Wolfgang Hiddemann**: Supervision; Resources. **Klaus H. Metzeler**: Supervision; Methodology; Conceptualization; Resources; Writing—review and editing. **Philipp A. Greif**: Methodology; Supervision. **Michael Heuser**: Resources; Supervision. **Daniel B. Lipka**: Methodology; Supervision. **Marion Subklewe**: Supervision; Resources; Methodology. **Sebastian Vosberg**: Supervision; Formal analysis; Visualization. **Tobias Herold**: Conceptualization; Writing—review and editing; Project administration; Supervision; Resources; Data curation; Formal analysis. **Karsten Spiekermann**: Conceptualization; Funding acquisition; Writing—review and editing; Project administration; Supervision; Resources. **Irmela Jeremias**: Conceptualization; Funding acquisition; Writing—original draft; Writing—review and editing; Project administration; Supervision; Methodology; Visualization.

## CONFLICT OF INTEREST STATEMENT

T.H. has received travel support from Jazz Pharmaceuticals and honoraria for speakers from Astellas Pharma and participates on the Advisory Board of Servier and Jazz Pharmaceuticals. All other authors declare no conflict of interest.

## ETHICS STATEMENT

BM and peripheral blood (PB) samples from AML patients were collected for diagnostic purposes. Written informed consent was obtained from all patients or from their legal representatives in case patients were minors. The study was performed in accordance with the Helsinki Declaration of 1975, as revised in 2013, and with the ethical standards of the responsible committees on human experimentation (Research Ethics Boards of the medical faculty of LMU Munich, #068‐08, 222‐10, and 20‐739). Animal trials were performed in accordance with the ethical standards of the official committee on animal experimentation.

## FUNDING

The work was supported by grants from the European Research Council (Consolidator Grant 681524) and the Deutsche Forschungsgemeinschaft (DFG, German Research Foundation)—537701335 (Reinhart Koselleck project) (533056198 (SFB1709/12025)) and SFB 1709/1 2025—533056198; a Mildred Scheel Professorship (70114478, 70115044) by German Cancer Aid; Bettina Bräu Stiftung and Dr. Helmut Legerlotz Stiftung (all to IJ), by funding from the Deutsche José Carreras Leukämie‐Stiftung (DJCLS 15 R/2021, DJCLS 02 R/2023, and DJCLS 16R/2021 to I.J. and P.M.), by grants 70114478 and 70115044 from Deutsche Krebshilfe, grant R/2021 from DJCLS, and grant No. 101136502 under the European Union's HORIZON‐MISSION‐2023‐CANCER‐01 program (all to MH), and by the German Childhood Cancer Foundation, grant No. DKS2025.10 (to M. Schönung and D.B.L.). M. Schönung was supported by the Joachim Herz Foundation (Add‐on Fellowships for Interdisciplinary Life Science). Open Access funding enabled and organized by Projekt DEAL.

## Supporting information


**Supplemental Table 1: All samples and analyses**.

Supplemental Table 3: Publications with *PDX models.

Supplemental Table 2: Patient characteristics of cohort 1. Supplemental Figure 1. Primary engraftment and robust serial engraftment are confined to a minority of PDX models; related to Figure 1. Supplemental Figure 2. *PDX models represent aggressive diseases of diverse AML subgroups and resemble primary AML cells better than cell lines; related to Figure 2. Supplemental Figure 3. *PDX models resemble primary AML cells; related to Figure 3. Supplemental Figure 4. *PDX models allow a wide range of ***ex vivo*** **and** ***in vivo*** **experiments, including long‐term** ***in vivo*** **treatment trials, related to Figure 4**.

## Data Availability

scrbSeq and methylome data are available at GEO under accession numbers GSE307747 and GSE308997, respectively. PDX models can be accessed via CancerModels.org, and detailed information is provided in Supporting Information S1: Table 1. Cells will be shared with interested researchers upon request. For original data and sharing of PDX models, please contact binje.vick@helmholtz-munich.de.
