# Supplementary material for: A platform of serially transplantable AML PDX models covering subgroups for which no cell lines exist
Source: Hemasphere. 2026 Oct 5;10(10):e70497. doi: 10.1002/hem3.70497 (PMC13637652; doi:10.1002/hem3.70497)
Supplement: Supplementary file 1 [file HEM3-10-e70497-s003.docx]

**A platform of serially transplantable AML PDX models covering subgroups for which no cell lines exist**

Running title: Serially transplantable and robust AML PDX models

**Supplemental Material**

Supplemental Materials and Methods	page 2

Supplemental References	page 11

Supplemental Tables	page 13

Supplemental Figures	page 14

**Supplemental Materials and Methods**

**Sample Cohorts**

Primary adult patient samples of **cohort 1** (n=74) were collected between 2010 and 2019 in the Department of Internal Medicine III, Ludwig-Maximilians-Universität (LMU), Munich, Germany. Details on clinical parameters such as treatment regimens, survival, mutational status etc. were available for samples of this cohort.

Primary adult patient samples of **cohort 2** (n=27) were collected between 2012 and 2019 in the Department of Hematology, Oncology and Cancer Immunology, Charité, Berlin, Germany (n=9); Department of Medicine III, Technical University of Munich (TUM), Munich, Germany (n=6); Division of Hematology & Oncology, Department of Medicine, University of Pennsylvania, Philadelphia, USA (n=5); Department of Hematology and Oncology, Freiburg University Medical Center, Albert-Ludwigs-University of Freiburg, Breisgau, Germany (n=5); and Department of Internal Medicine I, University Hospital Carl Gustav Carus, University of Technology, Dresden, Germany (n=2).

Patient-derived xenograft (PDX) samples of **cohort 3** (n=22) were established at Inserm, Cancer Research Center of Toulouse, Toulouse, France (n=9)^1^; Department of Hematology and Oncology, Freiburg University Medical Center, Albert-Ludwigs-University of Freiburg, Breisgau, Germany (n=2); Department of General Pediatrics, Hematology and Oncology, University Children's Hospital, Tuebingen, Germany (n=5)^2^; Josep Carreras Leukemia Research Institute, Department of Biomedicine, School of Medicine, University of Barcelona, Barcelona, Spain (n=4)^3^ or from the PRoXe consortium, Boston, USA^4^ (n=2) and originated from adult or pediatric patients.

Primary pediatric patient samples of **cohort 4** (n=7) were collected between 2001 and 2014 in children’s hospitals throughout Germany; PDX models thereof were established in NSG mice at Hannover Medical School and University Hospital Halle (Saale), Germany.

Primary adult patient samples of **cohort 5** (n=32) were collected between 2013 and 2023 and PDX models thereof were established in pre-irradiated NSG or NSGS mice at Hannover Medical School, Germany ^5,6^.

**Mouse housing**

All animal trials were performed in accordance with the current ethical standards of the official committee on animal experimentation (written approval by Regierung von Oberbayern, tierversuche@reg-ob.bayern.de; 55.2-1-54-2531.6-10-10, 55.2-1-54-2531-95-10, 55.2-2532.Vet_03-16-56, 55.2-2532.Vet_02-16-7, 55.2-2532.Vet_02-20-159, 55.2-2532.Vet_03-21-9) and according to ARRIVE guidelines^7^. Mice were kept in animal rooms of the Laboratory Animal Breeding and Husbandry Unit of Helmholtz Munich under specified pathogen-free (SPF) conditions with a 12/12‑hour light cycle. The animal rooms of the barriers were fully air-conditioned with a temperature of 20-24°C and 45‑65% humidity according to Annex A of the European Convention 2007/526 EC. The maximum stocking density of the cages corresponds to Annex III of the 2010/63 EU. The cages were constantly filled with structural enrichment and the animals had unlimited access to food and water. During the experiment, mice were kept in individually ventilated cages (IVC). Hygiene monitoring was carried out at least quarterly in accordance with the FELASA recommendation: In the animal housing areas equipped with IVC systems, exhaust dust from the IVC ventilation units was tested for all FELASA-listed pathogens by PCR. Mice were examined daily, and health status was scored once a week (mice used for cell amplifications) or daily (mice receiving therapy).

**Isolation of primary AML blasts**

Human mononuclear cells were isolated from heparinized bone marrow (BM) aspirates or peripheral blood (PB) by density gradient centrifugation (Ficoll-PaqueTM PLUS, GE Healthcare, Uppsala, Sweden). Cells were washed twice in phosphate buffered saline (PBS; Life Technologies, Darmstadt, Germany) and either viable frozen in 90% FCS (Biochrom AG, Berlin, Germany) / 10% DMSO (Sigma-Aldrich, St. Louis, MO, USA) or used for transplantation. Frozen cells were thawed according to published protocols^8^.

**Monitoring engraftment of cells**

To monitor engraftment and growth of human cells in NSG mice, 50 µl of PB was repetitively collected by tail vein aspiration every other week starting from week four after cell injection. Depending on growth kinetics, sampling frequency was increased or decreased. BM was isolated after sacrifice of mice (see below). PB and BM were analyzed by flow cytometry after staining with antibodies against human CD45 (APC Mouse Anti-Human CD45 Clone HI30, BD Biosciences) and human CD33 (PE Mouse Anti-Human CD33 Clone WM53, BD Biosciences). Antibodies were added to 50 µL of PB or BM cell suspension, and incubated for 30 min. For PB, FACS lysing solution (BD Biosciences, Heidelberg, Germany) was added afterwards, and cells were incubated for another 15 min at room temperature. Cells were washed twice with PBS. Flow cytometry was performed with a BD LSRFortessa (BD Biosciences) using FACS Diva software (BD Biosciences). Flow cytometry data was analyzed using FlowJo software (TreeStar Inc., Ashland, OR, USA). Percentage of hCD45+ hCD33+ cells were analyzed within the “lymphocyte gate” in FSC/SSC. Positive engraftment was defined as >1% hDC33+ cells in the BM within 20 weeks after transplantation.

**Experimental end-points**

The majority of mice used for PDX cell amplification were sacrificed at end-stage leukemic disease (more than 50% leukemic cells within PB or when mild clinical signs of illness appeared, e.g., rough fur, mild hunchback, or slightly slowed movement); few mice developed moderate or severe symptoms (e.g. reduced motility, lameness, increased breathing frequency). Despite close monitoring of mouse health status by daily examinations and flow cytometry of PB, individual mice were found dead, either due to high leukemia cell engraftment, or due to leukemia-unrelated reasons (n=21, 1% of mice included in the study). If 20‑30 weeks after cell injection no human cells were detectable in PB and no clinical signs of illness arose, mice were sacrificed, and BM was analyzed by flow cytometry for human cell engraftment. Mice showing leukemia-unrelated illness or peculiarities were sacrificed and excluded from the studies. In experimental trials, specific endpoints were defined (i.e. defined bioluminescence signal). Mice which died in inhalation narcosis were excluded from further analyses. Mice showing therapy-related toxicity (i.e. loss of body weight beyond 15%) were sacrificed. Mice were sacrificed by exposure to CO_2_ or cervical dislocation.

**Recovering PDX cells from mice and serial transplantation**

PDX cells were reisolated from bones and spleen if this organ was macroscopically enlarged. Femurs, tibiae, hips, sternum and spine were crushed using a mortar and pistil in 10 ml PBS and filtered through a 70 µm cell strainer (EASYstrainer, Greiner Bio-One, Frickenhausen, Germany). Cell pellet was incubated with red blood cell lysis buffer (Sigma Aldrich) for 10 minutes and washed three times in PBS. Spleens were smashed through a 70 µm cell strainer and ficollized. Cells were counted with Trypan-blue exclusion using a Neubauer counting chamber, analyzed by flow cytometry, and frozen in 90% FCS / 10% DMSO (until 2022) or in Bambanker (Nippon Genetics, Düren, Germany) in a density of 5E6‑1E7 cells/ml and stored at ‑80°C or liquid nitrogen for long-term storage. Furthermore, 1E5 to 5E6 PDX cells were reinjected into next recipient mice for expansion or *in vivo* trials. Experimental mice were transplanted either with cells isolated freshly from donor mice, or with frozen/thawed cells from previous donor mice. Specimens were named with a specific nomenclature to be able to trace back donor cells and re-passaging steps.

**Authentication of passaged PDX cells**

Accuracy of model identity was regularly verified by repetitive finger printing using PCR of mitochondrial DNA^9^. Primary patient cells (if available) or early passage PDX cells were used as reference sequence.

**Lentiviral transduction and enrichment of genetically engineered PDX cells**

Lentiviruses were produced with a third-generation lentiviral system as published^10^. In this study, AML PDX cells were genetically engineered (GE) to express enhanced firefly luciferase and mCherry as marker fluorochrome, Cre‑ER^T2^ and mCherry (Plasmids #104833 or #178185, Addgene, Watertown, Massachusetts, USA), or Cas9 and GFP^11-13^.

PDX cells were isolated from donor mice and resuspended in StemPro‑34 medium (Thermo Fisher Scientific Waltham, MA, USA) supplemented with 1% L-Glutamine, 1% Penicillin/Streptomycin, 2% FCS (all Gibco, San Diego, CA, USA), 10 ng/ml hrFLT3L (R&D Systems, Minneapolis, MN, USA), 10 ng/ml hrSCF, 10 ng/ml hrTPO, and 10 ng/ml hrIL3 (all Peprotech, Rocky Hill, NJ, USA) as proposed recently^14^ at a density of 1E7 cells per ml, and kept at 37°C, 5% CO_2_. Cells were transduced overnight with lentiviral constructs in the presence of 8 µg/ml polybrene (Sigma-Aldrich). Transductions were performed at low MOI to guarantee single integrations. After 24 h to 48 h, cells were washed three times with PBS and injected into recipient mice for amplification. At end-stage leukemic disease, mice were sacrificed and PDX cells were isolated from murine BM or spleen. GEPDX cells were enriched by sorting for mCherry-positive or GFP-positive cells on a FACSAria II (BD). Enriched GEPDX cells were re-injected into mice for amplification. All therapy trials were performed with GEPDX cells purified to levels beyond 90% mCherry+ cells.

**Bioluminescence in vivo imaging (BLI)**

Bioluminescence was measured using an IVIS Lumina 2 (Perkin Elmer) to repetitively visualize outgrowth of luciferase+ GEPDX cells in living mice. Mice were anesthetized by isoflurane inhalation. For cells expressing a recombinant codon-optimized form of firefly luciferase, 150 mg/kg D-Luciferin (BIOMOL GmbH, Hamburg, Germany) was injected into the tail vein. Images were taken immediately for 30 sec or up to 2 min using a field of view of 12.5 cm with binning 8, f/stop 1 and open filter setting. Images with more than 1000 counts were considered within the linear range; saturated images were repeated with reduced exposure time or reduced f‑stop.

**Quantification of BLI pictures**

Quantification of light emission was performed using the Living Image software (Caliper Life Sciences, Mainz, Germany). A region of interest (ROI) covering the whole mouse was used and total flux was determined.

**Cultivation of PDX cells and *ex vivo* therapy trials**

PDX cells were isolated from donor mice, resuspended in StemPro‑34 medium (see above) and were kept at a density of 2E6 cells per ml. Viability and cell concentration were checked regularly by flow cytometry and cell counting. 50% of medium was replaced once to twice per week, depending on consumption. For *ex vivo* therapy trials, cells were seeded into white 96-well-plates (Greiner Bio-One), with 40,000 cells and 100 µl of medium per well, and treated with several concentrations of azacitidine, decitabine, cytarabine, daunorubicin (all Biozol Diagnostica, Hamburg, Germany), a combination of cytarabine and daunorubicin at a molar ratio of 5:1, or venetoclax (SelleckChem) in triplicates. After 72h, 40 µl of Cell Titer Glo reagent (Promega, Walldorf, Germany) was added, and luminescence was measured by a plate-reader 10 minutes later. Relative luminescence signal was calculated compared to DMSO-treated control cells. IC50 values were calculated with GraphPad Prism.

**Short-term phase II-like therapy trials**

Treatment with cytarabine (AraC) and DaunoXome (DNX): *PDX cells were injected into groups of mice (between 5E5 and 2E6 luc^+^ cells/mouse). Tumor burden was regularly monitored by BLI. When BLI signals reached a total flux between 1E8 and 4E9 Photons/second, mice were either treated with PBS, AraC 100 mg/kg (d1-4 and d8-11, i.p.) and DaunoXome 1 mg/kg (d1,4,8,11, i.v.), or AraC 100 mg/kg (d1-4 and d15-18). Effect on tumor burden was monitored by BLI and quantified. Relative tumor burden compared to treatment start was calculated by dividing BLI values by values of each individual mouse at d0. Response rates were categorized by comparing tumor burden at the end of treatment to tumor burden at treatment start; progressive disease (PD): relative tumor burden >200%; stable disease (SD): relative tumor burden between 50% and 200%; partial response (PR): relative tumor burden between 5% and <50%, similar to response rates defined for mouse models of solid tumors^15^.

**Calculation of tumor growth rate by analyzing doubling time of BLI signals**

To calculate growth rate of tumor cells, an exponential trend line was adjusted within the logarithmic growth phase of PDX cells in individual mice (total flux between 1E7 and 1E10 Photons / second). BLI doubling time in days was calculated by dividing ln(2)/k, with k being the exponential growth coefficient u(t)=‑c*e^k*t^.

**Blood cell count**

Blood cell count was performed on an XN9000 automated analyzer (Sysmex, Norderstedt, Germany). Analyses were performed in a 1:7 dilution of EDTA-anticoagulated whole blood with Cellpack (Sysmex).

**Sequencing**

**DNA and RNA preparation**

gDNA was prepared using the QIAamp DNA Mini Kit (Qiagen), RNA was prepared using the Direct-zol RNA Miniprep (Zymo Research, Freiburg, Germany). Concentration of dsDNA was determined via Qubit dsDNA BR Assay Kit (Life Technologies); concentration of RNA was determined via NanoDrop, quality of RNA was determined via Agilent RNA 6000 Nano Kit (Agilent Technologies, Santa Clara, CA, USA).

**Targeted Sequencing of recurrently mutated genes**

68 genes recurrently mutated in myeloid malignancies were sequenced using a targeted amplicon-based enrichment assay (Haloplex, Agilent, Boeblingen, Germany) as previously described^16^.

**Low coverage whole genome sequencing and data analysis**

DNA was prepared for whole genome sequencing using SureSelect WGS kit (Agilent Technologies, Santa Clara, CA, USA). Libraries were 100 bp paired‑end sequenced using a NovaSeq 6000 sequencer (Illumina, San Diego, CA, USA). Median coverage was 1.6-fold.

**RNA sequencing and data analysis**

RNA was prepared using TruSeq Stranded Total RNA (Agilent Technologies). Libraries were 100 bp paired-end sequenced using a NovaSeq 6000 sequencer (Illumina, San Diego, CA, USA).

**Analysis of copy number variants (CNVs) from methylome**

CNVs from methylation data were inferred with the conumee2 package (v2.1.2) in R (v4.4.2). Raw IDAT files were processed using minfi (v1.52.1). Reference control sample of normal tissue was obtained from the minfiDataEPIC(v1.32.0). Processing followed documentation^17^. Probe-level annotations were generated with a minimum bin size of 500 kb and minimum probe count of 15.

**Analysis of CNVs from lcWGS**

CNVs from lcWGS were inferred using QDNAseq (v1.42.0/ R v4.4.2)^18^ and ichorCNA (v0.3.2)^19^, following each tools documentation. A bin size of 500 kb was used in both methods.

**CNV calling**

CNV calls from methylation data were considered high evidence if detected with a log_2_ ratio ≥ 0.5 and confirmed by lcWGS data with a log_2_ ratio of ≥ 0.5 based on one of the two tools plus ≥ 0.2 based on the second tool (QDNAseq or ichorCNA). CNV calls were considered low evidence if not supported by lcWGS data.

In lcWGS we distinguish between high- and low-quality data. If the standard deviation of log_2_ ratios in lcWGS data was ≥ 0.5 and in methylation data ≤ 0.25, samples were considered low quality. Data from samples AML‑573, AML‑346, AML‑640, and AML‑372 was considered low quality.

CNV calls from high quality lcWGS data were considered high evidence if detected with a log_2_ ratio ≥ 0.5 by at least one of the two tools (QDNAseq or ichorCNA), a log_2_ ratio of ≥ 0.2 in the second tool plus supported by methylation data with a log_2_ ratio ≥ 0.2. Calls were considered low evidence if not supported by methylation data but detected using QDNAseq and ichorCNA, both with a log_2_ ratio ≥ 0.5.

CNV calls from low quality lcWGS samples were only considered if detected with a log_2_ ratio ≥ 0.5 in both tools (QDNAseq or ichorCNA) and supported by a log_2_ ratio ≥ 0.4 in methylation data.

Calls are required to span at least two consecutive 500 kb bins.

**Analysis of fusion transcripts**

Fastq files were processed with the RIFTT pipeline (https://github.com/pkerbs/RIFTT), providing an evidence level in the range 1‑6. Known fusions, that are listed in ChimerDB (here called predescribed), were considered if evidence level was five or six. Unknown fusions, without a corresponding entry in this database (here called undescribed), were considered if evidence level was six.

**Gene expression profiling**

Bulk RNA sequencing was performed as described in detail in Janjic et al.^20^. Gene expression data were processed with the R packages *limma* (version 3.44.3) and *edgeR* (version 3.30.3). Samples with a library size of less than 500,000 were excluded from the analysis. Only genes with more than one read per million in at least 10% of samples were included.

**Methylome**

Methylation data was preprocessed with the R package *minfi* (version 1.50.0). Probes not mapped to a genomic region, probes measuring SNPs and non-CpG methylation, probes with SNPs and probes with missing values were removed from the analysis.

**Clinical data sets**

Data generated with AML PDX models were compared to the following data sets: AML‑CG‑1999 (n=864)^21^, AML-CG‑2008 (n=274)^22^, AMLHD98A (n=557)^23^, AMLHD98B (n=160)^24^, AML‑SG07‑04 (n=724)^25^, AML-CG Registry (n=271)^26^, primary AML samples of adult patients at relapsed disease of BEAT cohort (n=53)^27^, primary AML samples of adult patients at initial diagnosis or relapse CN cohort (n=50)^28^, AML cell lines generated from adult patients (n=54), eventually focusing on models with > 500 articles on Google Scholar as of March 2025 (n=19; AML‑14, EoL‑1, HEL, HL‑60, KASUMI‑3, KG‑1, Me‑1, ML‑2, MOLM‑13, MONO‑MAC‑6, NOMO‑1, OCI‑AML‑2, OCI‑AML‑3, OCI‑AML‑5, SKM‑1, SKNO‑1, TF‑1, U‑937, UT‑7)^29^.

**Prediction analysis for microarrays (PAM) analysis**

Samples from the AML‑CG‑1999 and AML‑CG‑2008 study (training set) were used to build a classifier for genes mutated in at least 20% of PDX models. For each gene, the prediction analysis for microarrays (PAM)^30^ was used to build a model that classifies samples into a non-mutated or a mutated group. The classifier was then tested on PDX models and human samples from the AML-CG Registry. To evaluate the performance, the true positive (TPR), true negative rate (TNR), positive predictive (PPV) and negative predictive value (NPV) were calculated.

The gene expression training set contained 218, and the methylation training set contained 209 samples. The classifiers were tested on 271 gene expression and 224 methylation samples from the AML-CG Registry, and on 18 gene expression and 19 methylation PDX models.

**DNA methylation-based disease characterization**

Epigenetic classification of PDX models was performed as described recently^31^.

**Flow cytometry of surface antigens**

Surface expression of CD14 (clone: RMO52), CD33 (D3HL60.251), CD34 (581), CD38 (LS198.4.3), CD64 (22), CD65 (88H7), CD117 (104D2D1), CD123 (7G3), CD135 (SF1.340), HLA-DR (Immu-357, all Beckman Coulter, Marseille, France), CD7 (M-T701), CD13 (L138), CD56 (NCAM16.2, all BD Biosciences, Milpitas, CA, USA), CD11b (ICRF44, Biolegend), CD15 (W6D3, both Biolegend, San Diego, CA, USA) was assessed on a Navios flow cytometer (Beckman Coulter, Krefeld, Germany).

**Illustrations**
Schemes were created in BioRender. Klein, F. (2025) <https://BioRender.com/ffj84eg>

**Supplemental References**

1.	Farge T, Saland E, de Toni F, et al. Chemotherapy-Resistant Human Acute Myeloid Leukemia Cells Are Not Enriched for Leukemic Stem Cells but Require Oxidative Metabolism. *Cancer Discov*. 2017;7(7):716-735.

2.	Woiterski J, Ebinger M, Witte KE, et al. Engraftment of low numbers of pediatric acute lymphoid and myeloid leukemias into NOD/SCID/IL2Rcgammanull mice reflects individual leukemogenecity and highly correlates with clinical outcome. *Int J Cancer*. 2013;133(7):1547-1556.

3.	Diaz de la Guardia R, Velasco-Hernandez T, Gutierrez-Aguera F, et al. Engraftment characterization of risk-stratified AML in NSGS mice. *Blood Adv*. 2021;5(23):4842-4854.

4.	Townsend EC, Murakami MA, Christodoulou A, et al. The Public Repository of Xenografts Enables Discovery and Randomized Phase II-like Trials in Mice. *Cancer Cell*. 2016;29(4):574-586.

5.	Charles Cano F, Kloos A, Hebalkar RY, et al. XPO1-dependency of DEK::NUP214 leukemia. *Leukemia*. 2025;39(5):1102-1113.

6.	Hockendorf U, Dutta S, Kloos A, et al. Lymphotoxin alpha eradicates acute myeloid leukemia and simultaneously promotes healthy hematopoiesis in mice. *Sci Transl Med*. 2025;17(826):eadu3313.

7.	Percie du Sert N, Hurst V, Ahluwalia A, et al. The ARRIVE guidelines 2.0: Updated guidelines for reporting animal research. *PLoS Biol*. 2020;18(7):e3000410.

8.	Bonnet D. In vivo evaluation of leukemic stem cells through the xenotransplantation model. *Curr Protoc Stem Cell Biol*. 2008;Chapter 3:Unit 3 2.

9.	Hutter G, Nickenig C, Garritsen H, et al. Use of polymorphisms in the noncoding region of the human mitochondrial genome to identify potential contamination of human leukemia-lymphoma cell lines. *Hematol J*. 2004;5(1):61-68.

10.	Terziyska N, Castro Alves C, Groiss V, et al. In vivo imaging enables high resolution preclinical trials on patients' leukemia cells growing in mice. *PLoS One*. 2012;7(12):e52798.

11.	Bahrami E, Schmid JP, Jurinovic V, et al. Combined proteomics and CRISPR‒Cas9 screens in PDX identify ADAM10 as essential for leukemia in vivo. *Mol Cancer*. 2023;22(1):107.

12.	Carlet M, Volse K, Vergalli J, et al. In vivo inducible reverse genetics in patients' tumors to identify individual therapeutic targets. *Nat Commun*. 2021;12(1):5655.

13.	Vick B, Rothenberg M, Sandhofer N, et al. An advanced preclinical mouse model for acute myeloid leukemia using patients' cells of various genetic subgroups and in vivo bioluminescence imaging. *PLoS One*. 2015;10(3):e0120925.

14.	Wermke M, Camgoz A, Paszkowski-Rogacz M, et al. RNAi profiling of primary human AML cells identifies ROCK1 as a therapeutic target and nominates fasudil as an antileukemic drug. *Blood*. 2015;125(24):3760-3768.

15.	Eisenhauer EA, Therasse P, Bogaerts J, et al. New response evaluation criteria in solid tumours: Revised RECIST guideline (version 1.1). *European Journal of Cancer*. 2009;45(2):228-247.

16.	Metzeler KH, Herold T, Rothenberg-Thurley M, et al. Spectrum and prognostic relevance of driver gene mutations in acute myeloid leukemia. *Blood*. 2016;128(5):686-698.

17.	Daenekas B, Perez E, Boniolo F, et al. Conumee 2.0: enhanced copy-number variation analysis from DNA methylation arrays for humans and mice. *Bioinformatics*. 2024;40(2).

18.	Scheinin I, Sie D, Bengtsson H, et al. DNA copy number analysis of fresh and formalin-fixed specimens by shallow whole-genome sequencing with identification and exclusion of problematic regions in the genome assembly. *Genome Res*. 2014;24(12):2022-2032.

19.	Adalsteinsson VA, Ha G, Freeman SS, et al. Scalable whole-exome sequencing of cell-free DNA reveals high concordance with metastatic tumors. *Nat Commun*. 2017;8(1):1324.

20.	Janjic A, Wange LE, Bagnoli JW, et al. Prime-seq, efficient and powerful bulk RNA sequencing. *Genome Biology*. 2022;23(1):88.

21.	Buchner T, Berdel WE, Haferlach C, et al. Age-related risk profile and chemotherapy dose response in acute myeloid leukemia: a study by the German Acute Myeloid Leukemia Cooperative Group. *J Clin Oncol*. 2009;27(1):61-69.

22.	Braess J, Amler S, Kreuzer KA, et al. Sequential high-dose cytarabine and mitoxantrone (S-HAM) versus standard double induction in acute myeloid leukemia-a phase 3 study. *Leukemia*. 2018;32(12):2558-2571.

23.	Schlenk RF, Dohner K, Mack S, et al. Prospective evaluation of allogeneic hematopoietic stem-cell transplantation from matched related and matched unrelated donors in younger adults with high-risk acute myeloid leukemia: German-Austrian trial AMLHD98A. *J Clin Oncol*. 2010;28(30):4642-4648.

24.	Schlenk RF, Frohling S, Hartmann F, et al. Intensive consolidation versus oral maintenance therapy in patients 61 years or older with acute myeloid leukemia in first remission: results of second randomization of the AML HD98-B treatment Trial. *Leukemia*. 2006;20(4):748-750.

25.	Schlenk RF, Lubbert M, Benner A, et al. All-trans retinoic acid as adjunct to intensive treatment in younger adult patients with acute myeloid leukemia: results of the randomized AMLSG 07-04 study. *Ann Hematol*. 2016;95(12):1931-1942.

26.	Rausch C, Rothenberg-Thurley M, Dufour A, et al. Validation and refinement of the 2022 European LeukemiaNet genetic risk stratification of acute myeloid leukemia. *Leukemia*. 2023;37(6):1234-1244.

27.	Bottomly D, Long N, Schultz AR, et al. Integrative analysis of drug response and clinical outcome in acute myeloid leukemia. *Cancer Cell*. 2022;40(8):850-864 e859.

28.	Greif PA, Hartmann L, Vosberg S, et al. Evolution of Cytogenetically Normal Acute Myeloid Leukemia During Therapy and Relapse: An Exome Sequencing Study of 50 Patients. *Clin Cancer Res*. 2018;24(7):1716-1726.

29.	Pettirossi V, Venanzi A, Spanhol-Rosseto A, et al. The gene mutation landscape of acute myeloid leukemia cell lines and its exemplar use to study the BCOR tumor suppressor. *Leukemia*. 2023;37(2):473-477.

30.	Tibshirani R, Hastie T, Narasimhan B, Chu G. Diagnosis of multiple cancer types by shrunken centroids of gene expression. *Proc Natl Acad Sci U S A*. 2002;99(10):6567-6572.

31.	Schönung M, Türe M, Lajer P, et al. Hierarchical classification of hematologic malignancies using epigenetic and genetic information. *bioRxiv*. 2026:2026.2007.2002.735835.

32.	Zeller C, Richter D, Jurinovic V, et al. Adverse stem cell clones within a single patient’s tumor predict clinical outcome in AML patients. *Journal of Hematology & Oncology*. 2022;15(1):25.

**Supplemental Tables**

**Supplemental Table 1: All samples and analyses**

Separate excel file

**Supplemental Table 2: Patient characteristics of cohort 1**

| **Parameter** | **Value** | **Engrafted** | **Not engrafted** | ***p*-value** |
| --- | --- | --- | --- | --- |
|  |  | **(n = 31)** | **(n = 43)** |  |
| **Age** | Median  [range] | 55 [20 - 81] | 59 [21 - 81] | 0.5 |
| **Sex female** | n (%) | 22 (71) | 24 (56) | 0.23 |
| **Relapse** | n (%) | 15 (48) | 9 (21) | 0.022 |
| **WBC (G/L)** | Median  [range] | 21.2 [0.2 - 288] | 25.5 [0.1 – 233.0] | 0.97 |
| **Hb (g/dL)** | Median  [range] | 10.0 [5.2 – 14.4] | 9.3 [5.4 – 15.6] | 0.24 |
| **Platelets (G/L)** | Median  [range] | 46.0 [5.0 – 380.0] | 54 [7.0 – 190.0] | 0.25 |
| **Cytogenetically normal** | n (%) | 13 (57) | 23 (56) | >0.999 |
| **Complex karyotype** | n (%) | 3 (12) | 5 (12) | >0.999 |
| **FAB M0** | n (%) | 0 (0) | 1 (3) | 0.89 |
| **FAB M1** | n (%) | 9 (33) | 14 (37) |  |
| **FAB M2** | n (%) | 5 (19) | 5 (13) |  |
| **FAB M4** | n (%) | 9 (33) | 9 (24) |  |
| **FAB M5** | n (%) | 4 (15) | 8 (21) |  |
| **FAB M7** | n (%) | 0 (0) | 1 (3) |  |
| **ELN 2022 favorable** | n (%) | 3 (10) | 13 (30) | 0.11 |
| **ELN 2022 intermediate** | n (%) | 10 (32) | 11 (26) |  |
| **ELN 2022 adverse** | n (%) | 18 (58) | 19 (44) |  |

ELN: European Leukemia Net classification system, FAB: French-American-British classification system Hb: hemoglobin, WBC: white blood cells

Continuous variables were analyzed with the Mann-Whitney-U-Test, and the categorical variables with Fisher’s exact text.

**Supplemental Table 3: Publications with *PDX models**

Separate excel file

**Supplemental Figures**

**Supplemental Figure 1. Primary engraftment and robust serial engraftment are confined to a minority of PDX models; related to Figure 1.**

**A-D** Primary patients’ AML cells (2.5E5 – 2E7) were transplanted into one to eight male or female NSG mice via tail vein injection. Positive engraftment was analyzed by flow cytometry of peripheral blood and bone marrow and/or spleen at experimental end point. If at least one mouse showed positive engraftment, the sample was classified as engrafted.

**A,B** Patient samples of cohort 1 (n=74) were analyzed concerning factors influencing primary engraftment.

**A** Bar plots show positive engraftment rate according to disease status (initial diagnosis (ID) versus relapsed / refractory disease (R/R), left, *p=*0.026), *FLT3* mutation status (*FLT3* wildtype (wt) versus *FLT3*-ITD, middle, *p*=0.0034) and *BCOR* mutation status (wt versus mutated (mut), right, *p*=0.084), respectively. The *p*-values were calculated with Fisher's exact test.

**B** Kaplan-Meier graph depicting the overall survival (OS) probability stratified by positive (black) or negative (grey) engraftment of primary patient cells. Left: all samples (n=74); HR=1.73, 95%-CI [0.97, 3.07], *p*=0.063; middle: patients at ID (n=49), HR=1.55, 95%-CI [0.71, 3.], *p*=0.28; right: patients with R/R disease (n=25), HR=1.64, 95%-CI [0.66, 4.06], *p*=0.28.

**C** Pie charts displaying positive (black sector) or negative (grey sector) engraftment for primary samples of male or female patients (cohort 1 and 2) transplanted into male or female mice; only samples with known patient sex are included (n=89). Two samples were transplanted both in male and female mice and are therefore counted twice. Univariable analysis for the association between male sex (mouse) and primary engraftment, OR=0.27, *p*=0.0042.

**D** Positive engraftment (+, black dots) and negative engraftment (-, grey dots) are depicted for male and female mice of different age at time of injection. One dot represents one sample. n.s.: not significant as tested by two-sided unpaired t‑test with Welch´s correction.

**E** Related to Figure 1D. Distribution of samples collected from the different cohorts within Score 2 – Score 6.

**F-O** *PDX AML models (n=23) were serially passaged in NSG mice for amplification (donor mice), to generate genetically engineered models, and used for *in vivo* trials.

**F** Exemplary schematic phylogenetic tree of one model (AML‑491) re-transplanted several times into next recipient mice. Each small dot represents one donor mouse for amplification, each large dot an *in vivo* trial with several mice (n=6‑22), each line a re-transplantation step of PDX cells. Left part displays passaging of unmodified PDX cells, right part displays passaging of genetically engineered PDX cells (green). P: passage.

**G** *PDX models show indefinite growth *in vivo*. Median passaging time of each model at each re-transplantation step (passage) was calculated to depict total time each model had been growing in mice. Only mice used for PDX cell amplification which were sacrificed at end-stage leukemia were included in the analyses. Per re-passaging step and model, between one and 24 (median 5) mice were included. Grey bars at the end of each bar indicate the possibility for further re-transplantation steps. Samples are ordered according to total days of *in vitro* growth.

**H** Violin plot depicting the passaging time for all donor mice transplanted with Score 5 or Score 6 (bold) *PDX models; median indicated by dashed line and 25^th^ and 75^th^ percentile by dotted lines. Samples are ordered according to median passaging time.

**I** Passaging time was determined in donor mice serially transplanted with *PDX models and is depicted for each model concerning mouse sex (female or male), mouse age at time of cell transplantation (younger or older than 12 weeks), cell number injected (more or less than 0.5 million cells), cell condition (freshly isolated from donor mice or thawed), cells of low (P≤2) or high re-passaging step (P>2), and genetic engineering (unmodified or transgenic cells). Left: median values of each *PDX model. Right: all values of individual mice are included. Violin plots with median indicated by dashed line and 25^th^ and 75^th^ percentile by dotted lines. * *p*<0.05, ** *p*<0.01, *** *p*<0.001, **** *p*<0.0001 as analyzed by unpaired t-test with Welch’s correction (left), Fisher’s exact test (binary variables) or by Mann-Whitney-U‑test (continuous variables) for individual models (right).

**J** Positive engraftment rate (PER) was analyzed for mice injected with low or high cell numbers (more or less than 0.5 million cells, n.s.), freshly isolated or frozen/thawed cells (n.s.), cells at low or high passage numbers (n.s.), unmodified (no) or genetically engineered (yes) cells (*p*=0.014), and for cells injected into male or female mice (*p*=0.001) or into younger or older mice (below or above 12 weeks, n.s.). Dotted lines indicate PER between 75% and 100%. n.s. not significant, * *p*<0.05, ** *p*<0.01, **** *p*<0.0001 as analyzed by Fisher’s exact test (binary variables) or by Mann-Whitney-U‑test (continuous variables), both for individual models and across all models.

**K** Box plots showing percentage of engrafted mice transplanted with genetically engineered (yes) or unmodified (no) cells (left, n=23 models, *p*=0.042) and for male or female mice (right, n=22 models, *p*=0.001). Statistical significance was analyzed by Wilcoxon test.

**L** PER of cells transplanted into young mice (≤10 weeks), adult mice (10-20 weeks) or old mice (≥20 weeks at day of transplantation). n.s. not significant, * *p*<0.05 using univariate analysis. When applying a multivariate analysis, the difference was not significant (OR=0.53, *p*=0.20).

**M** Cell numbers of *PDX models re-isolated from bone marrow (BM) or spleen (only assessed when enlarged at the time point of sacrifice) of donor mice at end-stage leukemia are depicted.

**N** Highest value of hCD33+ cells in peripheral blood (pb) when mice had to be sacrificed due to mild clinical signs of illness.

**O** Percentage of mice per model with no or mild symptoms (black) or showing moderate or severe symptoms (white) at end-stage leukemic disease.

**Supplemental Figure 2. *PDX models represent aggressive diseases of diverse AML subgroups and resemble primary AML cells better than cell lines; related to Figure 2.**

**A-D *PDX models originate from AML patients with highly aggressive disease**

**A** Related to Figure 2A. Engrafted primary patient cells or PDX cells (n=62) were categorized into Score 1 to Score 6 as described in Figure 1B. Depicted are percentage of cases originating from ID (grey) or from R/R diseases (black).

**B** Related to Figure 2B. Kaplan-Meier curves showing the survival probability of all patients whose cells yielded *PDX models (Score 5/6, green) or did not meet the criteria of high score models (Score 2-4, blue). Log-rank test, *p*=0.00047.

**C** Related to Figure 2A. Left: percentage of models with wildtype *FLT3* (grey) or *FLT3-ITD* (black) are depicted (*p*=0.16). Right: percentage of models with wildtype *BCOR* (grey) or *BCOR* mutation (black) are depicted (*p*>0.999). The *p*‑values were calculated with Fisher's exact test.

**D** Related to Figure 2A. Kaplan-Meier curves showing the survival probability of patients with (n=369, red) or without (n=2210, black) mutations in *KRAS* or *WT1* or *ETV6*. Data collected from AML-CG‑1999 (n=864), AML-CG‑2008 (n=274), AMLHD98A (n=557), AMLHD98B (n=160), and AML‑SG07‑04 (n=724).

**E-G** Related to Figure 2C. Variant allele frequencies (VAF) of mutated genes detected via panel sequencing in primary patient cells (**E, G**) and in corresponding PDX cells (**E-G**). E: cohort 1 and 2; F: cohort 3; G: cohort 5. If VAF of *FLT3*-ITD was not determined, it was set to 50% (§). Data of some models have been published before (AML-372, AML-388 and AML-393: (V)^13^; AML-491 and AML-661: (Z)^32^).

**H,I** Related to Figure 2C. Copy Number Variants (CNVs) (**H**) and fusion transcripts (**I**) in *PDX models are depicted.

**J-L** related to Figure 2D.

**J** Data were analyzed as described in Figure 2D, showing factors represented both in *PDX models and cell lines (left) or factors not available in *PDX models (right). As analyzed cell lines all show a highly complex karyotype, chromosomal alterations never occur as isolated events. §: factors not analyzed in a respective dataset.

**K** Data were analyzed as described in Figure 2D, but all analyzed alterations are depicted and sorted according to relative frequencies in relapsed patients of BEAT cohort. §: factors not analyzed in a respective dataset.

**L** Relative frequencies of mutations and chromosomal alterations are depicted for primary AML samples at relapsed disease of BEAT cohort^27^ (n=53), primary AML samples at initial diagnosis of AML‑CG study^26^ (n=1138), primary AML samples at initial diagnosis or relapse of CN cohort^28^ (n=50), Score 3‑6 PDX models generated from adult patients (n=45) and AML cell lines generated from adult patients (n=54). Events are included which are present at a minimum of 5% in one of the five cohorts. Mutations are sorted according to relative frequencies in relapsed patients of BEAT cohort.

**M** Number of detected mutations of samples/models of the cohorts listed in Figure S2L.

**Supplemental Figure 3. *PDX models resemble primary AML cells; related to Figure 3.**

**A** Prediction Analysis of Microarrays (PAM) classifiers based on gene expression and DNA methylation profiling evaluated on *PDX models (n=19) and AML-CG Registry patient samples (n=224). Displayed are the true positive rate, true negative rate, positive predictive value and negative predictive value for the five genes mutated in at least 20% of PDX models. The predictive outcomes were largely comparable between primary patient samples and PDX models.

**B-D** related to Figure 3C. Primary patient samples at ID (n=20), primary patient samples at relapse (n=20), and *PDX models (n=23, green) were analyzed for cell surface marker expression via flow cytometry. Primary patient samples originated from an independent cohort and are not related to established PDX models.

**B** Violin plots depicting the percentage of antigen-positive cells within blast gate (upper) or mean fluorescence intensity (MFI, lower); median indicated by solid line and 25^th^ and 75^th^ percentile by dotted lines.

**C,D** Percent antigen-positive cells within blast gate (**C**) or mean fluorescence intensity (**D**) are depicted for each model.

**Supplemental Figure 4. *PDX models allow a wide range of *ex vivo* and *in vivo* experiments, including long-term *in vivo* treatment trials, related to Figure 4.**

**A-E** related to Figure 4A. Please also refer to Table S3 for a list of publications.

**A** Scheme illustrating functional trials performed with *PDX models. Luciferase-transgenic models have been used to determine (i) leukemia-initiating cell (LIC) / leukemia stem cell (LSC) frequencies by limiting dilution transplantation assays, (ii) re-engraftment capacity of *ex vivo* or *in vivo*-treated or genetically modified cells, (iii) dormant versus proliferating cells, or (iv) homing capacity into BM.

**B** Scheme illustrating molecular trials performed with *PDX models. Lentiviral transduction facilitates (i) genetic barcoding to analyze functional heterogeneity and marking of single cell clones; (ii) expression of different fluorochromes for competitive *in vivo* trials; (iii) expression of mutant or wildtype gene of interest (GOI), (iv) (inducible) knockdown of GOI; (v) CRISPR knockout of GOI, and (vi) *in vivo* CRISPR screens.

**C** Scheme illustrating preclinical therapy trials performed with *PDX models. Lentiviral transduction facilitates expression of enhanced firefly luciferase, enabling sensitive and repetitive disease monitoring and real-time follow-up of therapy response via bioluminescence imaging. Mice can be treated both short-term or long-term with either (i) chemotherapy, (ii) targeted therapy, (iii) immunotherapy such as CAR‑T cells, bi-specific T cell engager antibodies or immune activation, or (iv) antibody-drug-conjugates.

**D** PDX cells were genetically modified and enriched to express enhanced firefly luciferase for bioluminescence *in vivo* imaging (red), Cre-ER^T2^ for Tamoxifen-inducible expression of shRNAs and knockdown of target genes (blue) or split‑Cas9 for knockout of target genes (green).

**E** Thawed and freshly isolated cells of *PDX models have been used for diverse *ex vivo* trials, including phenotypic characterization, colony forming unit assays, *ex vivo* drug testing, *ex vivo* growth characteristics, knockdown or knockout approaches (Table S3 and unpublished). Due to reduced viability after thawing, most assays can only be performed with freshly isolated cells.

**F** PDX models of passage 6 (AML-388) or passage 4 (AML-491, AML-661) were isolated from donor mice at end-stage leukemia and cultivated *ex vivo*. Immunophenotype was analyzed directly after isolation and after 3, 7 and 14 days of culture. Then, 5E6 cells were retransplanted into next recipient mice. At end-stage leukemia, cells were re-isolated and immunophenotype was again analyzed directly after isolation. Percentage of antigen-positive cells within blast gate (left) or mean fluorescence intensity (MFI, right) are depicted.

**G-J** Related to Figures 4B-F.

**G** Complete blood count analysis of mice without PDX cell transplantation (ctrl, n=3), or of mice transplanted with *PDX AML-491 cells treated with PBS (n=4), cytarabine 50 mg/kg and DaunoXome 1 mg/kg (n=4), or cytarabine 100 mg/kg (n=3) four days after last treatment.

**H** Relative body weight of mice treated with 1^st^, 2^nd^ or 3^rd^ cycle of chemotherapy (grey boxes).

**I** Relative drop in leukemic burden under chemotherapy (1^st^, 2^nd^ or 3^rd^ cycle). Relative tumor burden after start of chemotherapy is depicted for 1^st^ (circle, n=24), 2^nd^ (square, n=7) and 3^rd^ (triangle, n=13) cycle of chemotherapy. Mean +/- standard deviation is shown. * *p*<0.01 as tested by two-sided unpaired t‑test with Welch´s correction. n.s. not significant.

**J** Panel sequencing was performed with re-isolated PDX cells. Doubling time did not correlate with NRAS status of the cells.

**K-M** Related to Figure 4G, AML‑372.

**K** Experiment was performed as described in Figure 4G; however, mice were treated with 2.5 mg/kg azacitidine (grey box). Data of Figure 4G is included as dotted line for comparison.

**L** Doubling time, measured by increase in BLI signal, is depicted for untreated mice (before azacitidine, n=4) or after stop of azacitidine therapy (n=5). Dots indicate values of individual mice. *** *p*<0.001 as tested by two-sided unpaired t-test with Welch´s correction.

**M** Relative weight of mice treated with azacitidine.

**N** related to Figure 4H. Total flux values (mean +/- standard deviation) of experiments described in Figure 4G are depicted. PD: progressive disease; PR: partial response; SD: stable disease. n.s.: not significant, ** *p*<0.01, **** *p*<0.0001, as tested by two-sided unpaired t-test.

**O-R** Primografts and luciferase-transgenic derivatives of late passage of *PDX models AML-388 (**O**), AML-393 (**P**), AML-491 (**Q**) and AML-661 (**R**) were transplanted into mice. At end-stage leukemia, cells were re-isolated, seeded in 96 well plates and treated with indicated drugs and concentrations. After 72h, cell viability was determined by CellTiterGlo analysis. IC50 values were determined with GraphPad Prism. n.s. not significant; * *p*<0.05, ** *p*<0.01, *** *p*<0.001, **** *p*<0.0001.
